# Bridging the gap in obesity research: A consensus statement from the European Society for Clinical Investigation

**DOI:** 10.1111/eci.70059

**Published:** 2025-05-15

**Authors:** Federico Carbone, Jean‐Pierre Després, John P. A. Ioannidis, Ian J. Neeland, Gabriella Garruti, Luca Busetto, Luca Liberale, Stefano Ministrini, Gemma Vilahur, Thomas H. Schindler, Maria Paula Macedo, Agostino Di Ciaula, Marcin Krawczyk, Andreas Geier, Gyorgy Baffy, Maria Felicia Faienza, Ilaria Farella, Nicola Santoro, Gema Frühbeck, Patricia Yárnoz‐Esquiroz, Javier Gómez‐Ambrosi, Emma Chávez‐Manzanera, Verónica Vázquez‐Velázquez, Jean‐Michel Oppert, Dimitrios N. Kiortsis, Paolo Sbraccia, Carmine Zoccali, Piero Portincasa, Fabrizio Montecucco

**Affiliations:** ^1^ Department of Internal Medicine University of Genoa Genoa Italy; ^2^ IRCCS Ospedale Policlinico San Martino, Genoa Genoa Italy; ^3^ Institut Universitaire de Cardiologie et de Pneumologie de Québec – Université Laval Québec Québec Canada; ^4^ VITAM – Centre de Recherche en santé Durable Centre intégré Universitaire de santé et de Services Sociaux de la Capitale‐Nationale Québec Québec Canada; ^5^ Department of Medicine Stanford Cardiovascular Institute, and Meta‐Research Innovation Center at Stanford (METRICS), Stanford University Stanford California USA; ^6^ Department of Epidemiology and Population Health Stanford Cardiovascular Institute, and Meta‐Research Innovation Center at Stanford (METRICS), Stanford University Stanford California USA; ^7^ Department of Biomedical Science Stanford Cardiovascular Institute, and Meta‐Research Innovation Center at Stanford (METRICS), Stanford University Stanford California USA; ^8^ Case Western Reserve University School of Medicine Cleveland Ohio USA; ^9^ Department of Cardiovascular Disease Harrington Heart and Vascular Institute Cleveland Ohio USA; ^10^ Department of Precision and Regenerative Medicine and Ionian Area (DiMePre‐J) University of Bari “Aldo Moro” Bari Italy; ^11^ Department of Medicine University of Padua Padua Italy; ^12^ Center for Molecular Cardiology University of Zurich Schlieren Switzerland; ^13^ Cardiology Department Luzerner Kantonspital Lucerne Switzerland; ^14^ Research Institute, Hospital de la Santa Creu i Sant Pau, IIB‐Sant Pau, IIB‐Sant Pau Barcelona Spain; ^15^ CiberCV, Institute Carlos III Madrid Spain; ^16^ Washington University in St. Louis, Mallinckrodt Institute of Radiology, Division of Nuclear Medicine, Cardiovascular Medicine Washington University School of Medicine St. Louis Missouri USA; ^17^ APDP – Diabetes Portugal Education and Research Center Lisbon Portugal; ^18^ iNOVA4Health, NOVA Medical School | Faculdade de Ciências Médicas, NMS | FCM Universidade Nova de Lisboa Lisbon Portugal; ^19^ Department of Gastroenterology, Hepatology and Transplant Medicine, Medical Faculty University of Duisburg‐Essen Essen Germany; ^20^ Laboratory of Metabolic Liver Diseases, Department of General, Transplant and Liver Surgery, Centre for Preclinical Research Medical University of Warsaw Warsaw Poland; ^21^ Interdisciplinary Amyloidosis Center of Northern Bavaria University Hospital of Würzburg Würzburg Germany; ^22^ Department of Internal Medicine II, Hepatology University Hospital of Würzburg Würzburg Germany; ^23^ Department of Medicine VA Boston Healthcare System, Harvard Medical School Boston Massachusetts USA; ^24^ Department of Medicine and Surgery LUM University Casamassima Italy; ^25^ Department of Pediatrics Yale University School of Medicine New Haven Connecticut USA; ^26^ Department of Medicine and Health Sciences “V. Tiberio” University of Molise Campobasso Italy; ^27^ Department of Endocrinology and Nutrition Cancer Center Clínica Universidad de Navarra (CCUN) Pamplona Spain; ^28^ IdiSNA (Instituto de Investigación en la Salud de Navarra) Pamplona Spain; ^29^ CIBERObn (CIBER Fisiopatología de la Obesidad y Nutrición) Instituto de Salud Carlos III Madrid Spain; ^30^ Instituto Nacional de Ciencias Médicas y Nutrición Salvador Zubirán Mexico City Mexico; ^31^ Department of Nutrition, Pitié‐Salpêtrière Hospital (AP‐HP), Human Nutrition Research Center Ile‐de‐France (CRNH IdF) Sorbonne University Paris France; ^32^ Atherothrombosis Research Centre, Faculty of Medicine University of Ioannina Ioannina Greece; ^33^ Department of Systems Medicine University of Rome Tor Vergata Rome Italy; ^34^ Renal Research Institute New York New York USA; ^35^ Institute of Molecular Biology and Genetics (Biogem) Ariano Irpino Italy; ^36^ Associazione Ipertensione Nefrologia Trapianto Renale (IPNET), c/o Nefrologia Grande Ospedale Metropolitano Reggio Calabria Italy

**Keywords:** body mass index, cardiovascular risk, management of obesity, metabolic dysfunction‐associated steatotic liver disease, metabolically healthy obesity, obesities, obesity, obesity definition, obesity transition, paediatric obesity, waist circumference

## Abstract

**Background:**

Most forms of obesity are associated with chronic diseases that remain a global public health challenge.

**Aims:**

Despite significant advancements in understanding its pathophysiology, effective management of obesity is hindered by the persistence of knowledge gaps in epidemiology, phenotypic heterogeneity and policy implementation.

**Materials and Methods:**

This consensus statement by the European Society for Clinical Investigation identifies eight critical areas requiring urgent attention. Key gaps include insufficient long‐term data on obesity trends, the inadequacy of body mass index (BMI) as a sole diagnostic measure, and insufficient recognition of phenotypic diversity in obesity‐related cardiometabolic risks. Moreover, the socio‐economic drivers of obesity and its transition across phenotypes remain poorly understood.

**Results:**

The syndemic nature of obesity, exacerbated by globalization and environmental changes, necessitates a holistic approach integrating global frameworks and community‐level interventions. This statement advocates for leveraging emerging technologies, such as artificial intelligence, to refine predictive models and address phenotypic variability. It underscores the importance of collaborative efforts among scientists, policymakers, and stakeholders to create tailored interventions and enduring policies.

**Discussion:**

The consensus highlights the need for harmonizing anthropometric and biochemical markers, fostering inclusive public health narratives and combating stigma associated with obesity. By addressing these gaps, this initiative aims to advance research, improve prevention strategies and optimize care delivery for people living with obesity.

**Conclusion:**

This collaborative effort marks a decisive step towards mitigating the obesity epidemic and its profound impact on global health systems. Ultimately, obesity should be considered as being largely the consequence of a socio‐economic model not compatible with optimal human health.

## Graphical abstract

This consensus highlights critical research gaps in obesity epidemiology, phenotypic heterogeneity and clinical management, calling for advanced methodologies, collaborative strategies and innovative policies to mitigate the global obesity epidemic and its socio‐economic impacts.

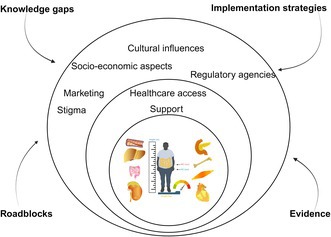

## 1 INTRODUCTION

Obesity is a serious relapsing chronic disease recognized as a leading cause of poor health globally. Like any chronic disease, obesity care requires a long‐term commitment, but its delivery is complex like any other. The paradigm of obesity is even being reframed from a pandemic perspective into a syndemic one, intertwined with the effects of climate change. In 2019, a dedicated Lancet commission clearly explained how feedback loops in governance, business, supply/demand and ecology serve as levers for the obesity syndemic.^1^ These macro‐systems govern the obesity syndemic, linking climate change—food and agriculture, urban design and land use are all contributors of obesity—with ‘obesogenic’ behaviours in micro‐systems (e.g. school, workplace, family). Somewhat paradoxical, individuals with obesity must commonly face a resilient form of social stigma. The prevailing view that obesity is a choice, entirely reversible through voluntary decisions to eat less and exercise more, extends from the workplace to educational setting and healthcare structures. This perspective ultimately exerts a negative influence on public health policies, access to treatments and research as well.^2^ Concerted efforts from broad stakeholders (e.g. healthcare providers, researchers, the media, policymakers and patients themselves) are warranted. While commendable initiatives have targeted specific aspects of the issue, an overarching goal is to identify gaps in the current scientific knowledge on obesity and collectively advocate for them. The responsibility of scientists covers indeed all levels. They are called to actively participate in policy monitoring and implementation through global frameworks and to be proactive in their mission by engaging in working groups, guidelines and position papers of international societies, all while integrating these efforts into their daily research work.^3^ This purpose well fits with the mission of the European Society for Clinical Investigation, which is reflected in this consensus statement. We here issue a call to action for all stakeholders to pledge their commitment to addressing research gaps in obesity research.

## 2 METHODOLOGY

The consensus development working group was convened by F.C. (project director) in collaboration with the European Society for Clinical Investigation. To systematically identify critical research gaps in obesity, we conducted a structured approach:

1. Comprehensive literature review: Each research gap was identified through an extensive review of current literature using major scientific databases (PubMed, Scopus, Web of Science). This review focused on analysing recent advancements, unresolved questions and existing limitations in obesity‐related research.
2. Identification and prioritization of key research gaps: Based on the literature review, eight core areas representing unmet research needs were selected: epidemiology, phenotypic heterogeneity, obesity‐related cardiometabolic risks, paediatric obesity, metabolic liver disease and clinical management of obesity. The selection was guided by their clinical relevance, scientific impact and potential for future research.
3. Expert panel formation and task leadership: The working group director (F.C.) and co‐chair (F.M.) appointed task leaders (Table 1), selecting them from the academic membership/council of the European Journal of Clinical Investigation and the European Society for Clinical Investigation. Task leaders were chosen based on their documented expertise and significant publication record in the respective fields. Each task leader was responsible for drafting an initial version of their section and assembling an expert panel covering endocrinology, nutrition, internal medicine, molecular biology, cardiology, gastroenterology, paediatrics, public health and health policy (Table 1).
4. Consensus and finalization process: The drafts from all task leaders were merged and circulated among the authors for critical revision. Feedback was integrated through an iterative process to ensure scientific accuracy and alignment with both clinical and public health priorities. The working group director (F.C.) coordinated the final preparation and submission of the consensus statement after achieving consensus and obtaining signed approval from all contributors.

**TABLE 1:** Summary of the expert panel.

Task	Task leader and expert panel	Affiliation
1. Gap in obesity epidemiology: understanding the obesity transition	**Federico Carbone** Fabrizio Montecucco John P.A. Ioannidis Gabriella Garruti	University of Genoa, Italy University of Genoa, Italy Stanford University, CA University of Bari “Aldo Moro”, Italy
2. From obesity to obesities—the gaps in considering its phenotypic heterogeneity	**Federico Carbone** Jean‐Pierre Després Ian J Neeland Luca Busetto	University of Genoa, Italy Université Laval, Canada University Hospitals Cleveland, OH University of Padova, Italy
3. Obesity and cardiovascular disease: gap in study design and patient assessment	**Luca Liberale** Stefano Ministrini Gemma Vilahur Thomas H. Schindler Maria Paula Macedo	University of Genoa, Italy University of Zurich, Switzerland Institut de Recerca Sant Pau, Barcelona, Spain Washington University in St. Louis, MO APDP – Diabetes Portugal, Education and Research Center, Lisbon, Portugal
4. Metabolic dysfunction‐associated steatotic liver disease (MASLD): Managing the whole by predicting the parts	**Piero Portincasa** Agostino Di Ciaula Marcin Krawczyk Andreas Geier, Gyorgy Baffy	University of Bari “Aldo Moro”, Italy University of Bari “Aldo Moro”, Italy Medical University of Warsaw, Poland University of Dusiburg‐Essen, Germany Harvard Medical School, Boston, MA
5. Metabolic complications of paediatric obesity: current knowledge and research gaps	**Maria Felicia Faienza** Ilaria Farella Nicola Santoro	University of Bari “Aldo Moro”, Italy University of LUM, Casamassima, Italy Yale university, CT
6. Management of obesities: clinical needs and current gap	**Gema Frühbeck** Patricia Yárnoz‐Esquiroz Javier Gómez‐Ambrosi Emma Chávez‐Manzanera Verónica Vázquez‐Velázquez Jean‐Michel Oppert Dimitrios N Kiortsis Paolo Sbraccia	University of Navarra, Spain University of Navarra, Spain University of Navarra, Spain Instituto Nacional de Ciencias Médicas y Nutrición Salvador Zubirán, Tlalpan, Mexico City, Mexico Instituto Nacional de Ciencias Médicas y Nutrición Salvador Zubirán, Tlalpan, Mexico City, Mexico Sorbonne University, Paris, France University of Ioannina, Ioannina, Greece Policlinico Tor Vergata, Rome, Italy
Appendix. Critical gaps in methodological and biostatistical approaches in current obesity research	**Carmine Zoccali**	Renal Research Institute, New York, NY

*Note*: Task leaders are highlighted in bold.

## 3 TASK 1. GAP IN OBESITY EPIDEMIOLOGY: UNDERSTANDING THE OBESITY TRANSITION

### 3.1 Background

The meanings attributed to fatness throughout human history broadly differ from contemporary ones. Only with the rise of global capitalism in the late 19th and early 20th centuries, obesity was no longer associated with opulence and wealth, but morally judged and stigmatized when slender bodies came to the forefront. Further decades were needed to define obesity as a disease, but without overcoming the associated stigma. Traditional epidemiological data described obesity as a condition more likely linked to female sex and low socio‐economic status. This view is still true globally but driven/biased by data from the high‐income countries, where data collection is more widespread.^4^ In low‐income countries, by contrast, obesity primarily affects middle‐aged wealthy women from urban regions. Projection of world obesity atlas of 2023 further estimates the prevalence of overweight/obesity to reach 51% worldwide by 2035 and 78% in the United States by 2030 (https://www.worldobesity.org/resources/resource‐library/world‐obesity‐atlas‐2023).

### 3.2 Summary of the evidence

The analysis of decade‐long changes and transitions of obesity trends is now underway. The term transition indeed encompasses demography, epidemiology and nutrition to describe changes in population health parameters such as the body mass index (BMI), and to provide insights into underlying determinants, outliers and future trends.^5^ When developed countries are considered, the rising tide of obesity epidemic was established between 1970s and 1980s. What set in motion this ‘push phase’ is matter of debate. The exponential rise in adult—and later childhood—obesity occurred simultaneously with a sharp increase in the consumption of fats and refined carbohydrates, due to their increasing affordability and easier access. The progressive introduction of obesogenic chemical compounds (e.g. plastics, fertilizers and additives) and other environmental contributors (e.g. fast foods, supermarkets and transportation facilities) made the slope steeper. Looking back, the ‘pull phase’ of previous decades served as flywheel. While urbanization and mechanization phenomena significantly reduced energy expenditure for daily living, there was a shift from a primarily plant‐based diet to one based on meat and ultra‐processed‐food items. These changes have promoted an unhealthy transition among people with higher disposable income, also referred to as ‘modernization theory’.^6^ The ‘obesity transitions theory’ provides an alternative view as categorizes those two phases as Stages 1 and 2, encompassing not only a historical but also a geographical perspective.^5^ While Vietnam is still at the start of obesity transition,^7^ other countries in South Asia and sub‐Saharan region are at Stage 1,^8^, ^9^ while members of the so‐called BRICS reached Stage 2.^5^, ^10^

### 3.3 Knowledge gap

At present, explaining the massive increase in obesity rates over the 20th century is a challenge. The modernization flow would expect a linear relationship between income and BMI, but this theory only holds true at low development levels. A long time ago, Szreter recognized that a rapid and over economic growth may actually result in worsening human health, thereby describing a U‐shape relationship between development and disease burden.^11^ Accordingly, the ‘obesity transitions theory’ accounts for this as a distinct phase (Stage 3), characterized by narrowing of sex differences coupled with a reversal of socio‐economic trends. While all forerunner countries in the process of obesity transition (i.e. those known as ‘Western’) have reached this stage in the last decade, subnational variation exists, reflecting socio‐economic factors and disparities. In those countries, high educational levels and the emergence of an active civil society has brought forward the democratization process. Ideally, one would like to see the implementation of regulatory policies to improve food security and consumer protection. However, an obesity paradox is generated in relative poverty conditions, where low‐income households are exposed to low food security due to limited time, knowledge, and resources to engage in healthy eating and exercise.^12^ While food insecurity is widely recognized as a key determinant of obesity, research in Europe has largely focused on its economic dimension, overlooking other structural factors such as food accessibility, food quality and production‐related barriers. Studies suggest that migrants and socio‐economically disadvantaged groups are disproportionately affected by food insecurity, yet there is a lack of comprehensive research on how these factors interact with obesity risk.^13^, ^14^ Addressing this gap is crucial to understanding the full spectrum of obesity drivers, particularly in populations where affordability is not the only limiting factor in dietary choices. Taking the progress in modernization for granted, the obesity transition in middle−/low‐income countries also points the finger at globalization/‘Westernization’. While economic globalization has been associated with unfair trade laws that saturate the mass market with low‐cost, unhealthy ultra‐processed foodstuffs, cultural globalization heavily enhances the appeal of Western lifestyle in the pursuit of ‘modernity’. From relative poverty to the bourgeoning middle class, globalization drivers expose people to a massive obesogenic environment.^10^, ^15^, ^16^, ^17^, ^18^, ^19^, ^20^ At the extremes of heterogeneity in obesity transition, there are insights on resilient modifiers. In East Asia, a depth cultural background influences food behaviour and markedly delayed the epidemic, even carrying propensity to a low BMI upon emigrating to western countries.^21^, ^22^ However, worldwide obesity trends in migrant populations provide a more complex picture. Migration and its associated socio‐environmental changes significantly—but differentially—influence obesity prevalence, particularly in middle‐ and high‐income countries. Despite regional heterogeneity, acculturation, economic integration and environmental factors contribute to a rising obesity burden, with one in four immigrants being obese and nearly half (49%) having a BMI ≥25.^23^ In Europe and North America, where immigrants often originate from low‐income regions, obesity rates are disproportionately higher in certain groups. In Norwegian and Spanish studies consistently highlight that longer residence duration correlates with increased obesity risk, both in childhood and pre‐pregnancy settings, with higher prevalence among individuals from Africa, Asia (except South Asia) and Eastern Europe.^24^, ^25^, ^26^ More broadly, the transition from undernutrition to obesity in immigrant communities is particularly striking and does not necessarily follow the traditional linear modernization model. Instead, this shift is shaped by globalization and sociocultural adaptation, reflecting complex interactions between dietary changes, urbanization and economic disparities, including an equal access to healthcare.^27^

Although the global obesity rates are alarmingly increasing, a trend towards stabilization is glimpsed in some developed countries since 2016. A flattening in the epidemic slope with a declining prevalence afterwards was then expected as hypothesized in the ‘obesity transitions theory’ as Stage 4, but without any validated explanation. More recent evidence generally challenges this view, but several concerns arise regarding the available data (Table 2).^28^, ^29^, ^30^, ^31^, ^32^, ^33^, ^34^, ^35^, ^36^, ^37^, ^38^ Real longitudinal data are limited, while many studies rely on meta‐analyses spanning decades. The estimated temporal trends are then arbitrarily categorized across decades without any rationale. Furthermore, all studies defined overweight/obesity according to BMI value, which is an easy but not sufficient measure of overweight/obesity. Furthermore, current projections for 2025 and beyond will undoubtedly be biased by the effects of the COVID‐19 pandemic.^39^ While imaging studies and those with physical activity data (Kaiser Permanente) have revealed that excess visceral adiposity and physical inactivity are largely responsible for the obesity‐COVID morbidity/mortality relationship,^40^, ^41^, ^42^ the associate increased of stress, anxiety and depression has prompted out unhealthy weight‐related behaviours, encompassing both eating patterns and sedentary habits globally. Furthermore, the COVID‐19 pandemic has further enhanced syndemic aspects related to social inequalities and food insecurity.^43^ Overall, a significant rise in obesity prevalence has been reported worldwide in association with the COVID‐19 pandemic (Table 3).^44^, ^45^, ^46^, ^47^, ^48^, ^49^, ^50^, ^51^, ^52^, ^53^, ^54^, ^55^, ^56^, ^57^

**TABLE 2:** Summary of contemporary studies analysing temporal trends in overweight/obesity prevalence.

Author	Year	Study design and time frame	Population characteristics	Findings
Kodaiara et al.^28^	2021	Meta‐analysis 1974–2020	Brazilian adult population	The prevalence of OW was 24.6% from 1974–1990 to 40.5% in 2011–2020. The prevalence of OW + OB increased from 33.5% to 52.5% between 1974–1990 and 2011–2020
Okati‐Aliabad et al.^29^	2022	Systematic review 2000–2020	Adults in the Middle East Countries	OB prevalence in the Middle East area remained steady between 2000–2006 and 2014–2020 (23%). During these time intervals, the prevalence of OW changed from 34.8 (95% CI: 32.4 to 37.5)
Bravo‐Saquicela et al.^30^	2022	Systematic review 1990–2016	Spanish pre‐school children (2–6 years old) and school‐aged children (6–13 years old)	Pooled estimate of OW and OB in pre‐school age increased from 23.3% in 1999–2010 to 39.9% in 2011–2021. In school‐aged children the prevalence increased from 32.3% in 1999–2010 to 35.3% in 2011–2021
Gao et al.^31^	2022	Data from nationally or regionally representative surveys 1980–2021	Pre‐school children (≤6 years old) and increased in school‐aged children (6–17 years old) in Mainland China and Hong Kong	In mainland China, OW and OB decreased in pre‐school children and increased in school‐aged children between 2010–2013 to 2015–2017 The prevalence of OW and OB in primary school students dropped in Hong Kong dropped from 21.4% in 2007–2008 to 17.6% in 2016–2017. The projections to 2030 suggest that the highest prevalence of OW and OB in preschool children will be in rural children
Vaidya et al.^32^	2022	Data from clinical treatment trials conducted by the SWOG Cancer Research Network 1986 and 2016	Patients registered to Phase 2 or Phase 3 treatment trials for obesity‐related cancers in US	Unadjusted OB rates increased from 23.5% (1986–1990) to 42.3% (2011–2016). There was an increasing linear trend in obesity with an OR of 1.23 for each 5‐year increase. The observed overall increasing trend in OB prevalence from 1999–2000 to 2015–2016 was greater in oncological than US adults
Anjana et al.^33^	2023	Cross‐sectional, population‐based survey (ICMR‐INDIAB) 2008–2020	Indian adults aged 20 years and older	The study reports a weighted prevalence of general and abdominal OB, which is higher in urban areas (40% vs. 23% and 52 vs. 34%, respectively). It also estimates a rise of general and abdominal OB to 254 and 351 million people in 2021
Kruger et al.^34^	2023	Meta‐analysis of 95 studies 1997–2022	South‐African children 6–19 years old	While OW and OB prevalence between 7 and 9 years old children were similar since 1999, among adolescents (10–19 years old) OW increased from 2002 to 2011 (16.9%–23.1%), 95% CI (21.5%, 24.9%). In meta‐regression analysis combined OW and OB increased over time in adolescents
Prvulović et al.^35^	2023	Meta‐analysis of 114 studies 2000–2009 vs. 2010–2020	Children 6–14 years old from European countries	Pooled estimate of OW and OB showed an increase in both sexes and all European regions except west countries
Dorner et al.^58^	2023	Three cross‐sectional Austrian surveys 2006/2007, 2014 and 2019	Austrian population aged ≥ 15 years 45,707	Prevalence of OB increased in both sexes in the study period (men 14%–20%, women 15%–18%; *p* < .001). In men, OB prevalence almost doubled from 2006/2007 to 2019 in subgroups of 15–29 years (5%–9.0%), unemployed (14%–28%) and from non‐EU/non‐EFTA countries (14%–26%). In women, the largest increase was found in subgroups of 30–64 years (16%–19%), those from non‐EU/non‐EFTA countries (20%–23%) and living in the federal capital Vienna (17%–20%)
Hu et al.^59^	2023	NHANES survey 1999–2020	US adults with diabetes 20 years of age or older 834–2073	The prevalence of overall obesity, obesity class II and obesity class III increased from 46.9%, 14.1% and 10.3% in 1999 to 2002 to 58.1%, 16.6% and 14.8% in 2015 to 2020, respectively. While the prevalence of patients who met control on BP, LDL‐c and glycaemia increased, it was worsen in OB patients
Fontbonne et al.^60^	2023	Obepi‐Roche surveys 1997–2020	French subjects aged ≥18 years 9598	The prevalence of excess weight was 47% (17% of OB subjects) with higher values in the north and east of France. Since 1997, OW was stable around 30%, whereas OB prevalence increased steadily at a rapid pace
Pham et al.^61^	2023	Vietnam STEPS (STEPwise approach to Surveillance) cross‐sectional Survey 2009–2015	Adult Vietnamese individuals 25–64 years old in 2009 18–69 years in 2015 14,706 to	Depending on different cut‐offs, OB prevalence increased from 1%–10% to 2%–16% in women and from 1%–10% to 2%–15% in men from 2009 to 2015. Similarly, the prevalence of abdominal obesity increased from 3%–31% to 8%–42% in women and from .3%–19% to .4%–25% in men
Schramm et al.^36^ Tolstrup et al.^37^	2023	Analysis of Danish Health and Morbidity Surveys 1987–2021	Danish adult	The prevalence of OB increased from 6.1% in 1987 to 18.4% in 2021. While there was no influence of income, the stratification for educational levels showed a less steep linear slope for higher educational level
Spanholi et al.^62^	2023	Survey according with WHO and IOTF criteria	Schoolchildren aged 7–14 years Sstate capital in southern Brazil 2963 to 1544	Over the whole period, the OB prevalence increased (WHO: 10%–14%; IOTF: 5%–8%), and especially in those from public schools (WHO: 11%–16%; IOTF: 6%–10%)
Bansal et al.^38^	2024	Meta‐analysis of 152 studies 1994–2023	Children aged ≤19 years from South Asian countries	Pooled OW prevalence showed slight variations around 12.5% Conversely, the prevalence of OB increased exponentially: from 4.4% before 2010 to 5.6% in 2010–2013, 7.5 2014–2018, until 2023. The prevalence of OB + OW in children also increased over time from 16.1% before 2010 to 21.1% in 2014–2018. However, there was a slight decrease in 2019–2023 (20.2%)
Lee et al.^63^	2024	Korea National Health and Nutrition Examination Survey 2005–2021	Korean older adults 27,902	While the increase of OW/OB prevalence was limited to men (29%–37%; *p* for trend < .001), the prevalence of self‐perceived OW/OB increase in both sexes: 19%–35% in men and 33% to 49% in women (*p* for trend < .001), regardless of their actual weight
Broadbent et al.^64^	2024	Health Survey for England and National Child Measurement Programme 1995–2019	UK children 2–15 years aged 65,253	Prevalence of childhood OW/OB increased from 26% to 32%, with the highest and fastest growth in those aged 11–15 years. Despite a plateau since 2004, there were over time inequalities widened by, household educational attainment, structure and ethnicity
Wu et al.^65^	2024	Two cross‐sectional health interviews and surveys 2011–2021	Individuals aged ≥35 years Rural China 8400 and 7572	The prevalence of obesity and central obesity increased substantially, from 5.9% and 50.2% to 12.1% and 58.0%, respectively (*p* < .01). These increasing rates existed in all subcategories, including sex, age, ethnicity, education, annual household income, access to medical services and socio‐economic position (*p* < .05)

*Note*: The search for studies was conducted up to March 2024 in the PubMed database. The terms identified for the PubMed search were ((((obesity [Title]) OR (overweigh t[Title])) AND (prevalence [Title])) AND (trend)) NOT (COVID[Title]) from 2023. The same terms were also used to identify systematic reviews and meta‐analysis in the period from 2021 onwards. The database searches were complemented with manual review of the reference lists of relevant articles. Finally, the search results were further refined by including studies that effectively provided time‐trend analyses.

Abbreviations: IOTF, International Obesity Task Force; OB, obesity; OW, overweight; PAHO, Pan American Health Organization.

**TABLE 3:** Summary of contemporary studies analysing trends in obesity prevalence during COVID‐19 pandemic.

Author	Year	Study design and time frame	Population characteristics	Findings
Anderson et al.^66^	2023	Meta‐analysis 2020–2021	74 articles including 3,213,776 subjects from 29 countries	The pooled mean difference in WC and BMI did not significantly increase in both children and adults. Similarly, the prevalence of obesity increased by 2% and 1%, respectively, whereas the pooled mean difference for waist circumference was 1.03 cm (95% CI −.08, 2.15) in children and adults
Hernandez‐Vasquez et al.^67^	2023	Demographic and Family Health Survey 2019–2021	Peruvian children under 5 years of age and their mothers 41,533	The prevalence of childhood OW/OB increased from 6.4% to 7.8% in 2021. The extent of increase was greater in female children <5 years old and mothers who self‐identified as non‐native, with secondary/higher education, belonging to the middle/upper wealth quintile and resided in an urban area and in the coastal region.
Siegel et al.^68^	2023	HER from Cincinnati Children's Hospital's Epic 2018–2021	US children aged 2–19 years 166,353	While OW/OB prevalence linearly increased (from 35% to 36.4%), the slope increases during the year of the COVID‐19‐related restriction (2020–2021) of 8.3%. Viewing at the percentile change in BMI, there was a significantly increasing trend (*p* < .001) during the pandemic year compared with the 2 years before
He et al.^44^	2023	Chengdu Positive Child Development (CPCD) survey 2019–2020	Chinese children from primary and secondary schools 5963	OW/OB prevalence increased from 9.2% and 8.6% to 10.5% and 10.6% during the pandemic (*p* < .001), respectively. While daily physical activity, sleep duration and sugar‐sweetened beverage consumption decreased, screen time increased. Ethnic minority, older age, less daily physical activity, reduced sleep duration and longer screen time were positively associated with obesity
Jenssen et al.^69^	2023	EHR data from Children's Hospital of Philadelphia's 2019–2022	US children who had at least one preventive visit during analytic periods 5–17 years old 153,667	OB prevalence significantly increased at the pandemic onset (OR: 1.2 [95% CI 1.21–1.25]) followed by a significant decrease (OR: .99 [95% CI .99–.99]). By December 2022, obesity had returned to pre‐pandemic levels, but persistent socio‐demographic disparities remained
Wuerdeman et al.^70^	2023	Military Health System Data Repository 2019–2021	Active‐duty US Army soldiers 191,894	There were significant changes across BMI categories during the pandemic, with a 5% growth and 27% change. More specifically, 26.7% of healthy classified soldiers shifted to OW, whereas 15.6% of OW shifted from to OB in the pandemic period. The highest increase was observed in female, ages 20–24, white, and Junior Enlisted soldiers
Park et al.^45^ Kim et al.^44^ Ban et al.^71^	2022 2023	Analysis of Korea Youth Risk Behavior Survey 2005–2020	Korean adolescents 12–18 years old 60,100	Although the 15‐year (2005–2020) trend changes in the prevalence of OB increase from 3.2%; in 2005–2008, to 8.6% in 2020, the slope decreased during the pandemic with a *β*diff, of −.027 (95% CI, −.028 to −.026)
Jeong et al.^72^	2023	Health checkup data from a University Hospital in Seoul, Korea 2016–2022	Korean individuals aged ≥18 years 276	While changes in body weight/fat percentage and BMI during the pandemic were not clinically significant, substantial decreased of physical activity (*p* < .001), WC increase (*p* < .001), and higher prevalence of MetS (from 20% to 31%; *p* < .001) occurred
Zaccardi et al.^73^	2023	Clinical Practice Research Datalink AURUM database 2017–2022	English individuals aged ≥18 years 273,529	Most individuals did not change BMI category post‐lockdown (66.4%–83.3%). When it occurred, the proportion was greater in women (12.6% vs. 9.5%) and < 45 years old individuals
Ochoa‐Moreno et al.^74^	2024	National Child Development Study English Longitudinal Study of Ageing 2006–2022	English children in their first year of formal schooling (age 4–5 years) and their last year of primary education (age 10–11 years) 17,415	Obesity prevalence among children aged 4–5 years increased by 45% in 2020/21 and then decreased to 10.1% in 2021/22, returning to the pre‐pandemic trend. In the age 10–11 years group, the prevalence of obesity and severe obesity, after a 4.5% increase in 2020/21, showed a decrease of 2.1% in 2021/22, remaining above the pre‐pandemic previous trend. The increase was twice as high in the most compared with the least deprived areas. With almost 56,000 additional OW/OB children, the study estimated an additional lifelong healthcare of £800 million
Cho et al.^75^	2024	Behavioral Risk Factor Surveillance System 2018–2021	US adults aged ≥26 years 1,107,673	Once adjusted for a secular trend and multiple covariates, states/territories with mandatory stay‐at‐home orders experienced a larger increase of OB prevalence (OR: 1.05 [95% CI 1.01–1.11]). This effect was greater in younger adults and individuals with lower school education level

*Note*: The search for studies was conducted from 2023 up to March 2024 in the PubMed database. The terms identified for the PubMed search were (((((body mass index [Title]) OR (obesity [Title])) OR (overweight [Title])) AND (prevalence)) AND (trend)) AND (COVID[Title]). The same terms were also used to identify systematic reviews and meta‐analysis in the same period The search was limited to articles that were written in English. The database searches were complemented with manual review of the reference lists of relevant articles. Finally, the search results were further refined by including studies that effectively provided time‐trend analyses.

Abbreviations: BM, body mass index; HER, Electronic Health Record; KNHNES, Korea National Health and Nutrition Examination Survey; OB, obesity; OW, overweight.

### 3.4 Implementation strategies and potential issues

The conceptual model for the obesity syndemic represents an inside‐out version of the socio‐ecological model. Like every natural system, obesity trajectories stand at the centre, while human layers overlay, moving outwards from macro to micro systems.^1^ Successfully addressing the conceptual and communicational challenge of connecting the global problem of obesity with macro systems (e.g. climate change) and our familial microsystems requires a coherent narrative. Firstly, we need to understand the drivers and modifiers of obesity transition even at subnational level. Within a syndemic view, geographical distribution critically differentiates individuals within the same countries. In the special case of India and mainland China, which comprise about 35% of the global population, their own country extension encompasses several geographical/ecological areas, each differently exposed to the climate change. There, the world's most densely populated urban areas still deal with endless rural spaces, where socio‐economic inequalities differently influence the obesity transition. Unfortunately, such estimates are lacking for most countries. It is expected that artificial intelligence will help in understanding the determinants of obesity prevalence in the near future. Already tested in data collected from 3142 counties in United States, machine learning models seem to explain 79% of the variance in obesity prevalence, with promising potential for future studies.^76^ AI can enhance individual‐level obesity research by leveraging wearable technology, electronic health records and real‐time dietary monitoring. AI‐driven models can predict long‐term obesity risk by analysing behavioural patterns, metabolic markers and lifestyle habits,^77^, ^78^ although their incremental benefits, if any, versus more standard methods, is still unknown. In addition, machine learning models enable macro‐level analyses. AI‐powered geospatial analyses may allow researchers to map obesity prevalence in relation to food deserts/fast food density, urbanization patterns and socio‐economic disparities, providing insights that inform public health and urban planning strategies.^79^, ^80^, ^81^, ^82^

As these methods continues to evolve, its application in obesity epidemiology may provide deeper insights into the dynamic interactions between individual behaviours, socio‐economic structures, and environmental factors. By harnessing AI‐driven predictive analytics, policymakers and public health experts can proactively design interventions tailored to specific populations, ensuring that obesity prevention strategies are data‐driven, targeted and responsive to the evolving global landscape of health disparities.

While a compelling history should create urgent action for implementing the existing policies, governance levers are still weak. Macro‐systems are monitored by global frameworks (e.g. WHO, United Nations or the independent International Network for Food and Obesity/NCDs Research, Monitoring and Action Support [INFORMAS]) through authoritative international guidelines, resolutions and treaties.^83^ However, the approach cannot be one‐size‐fits‐all because the condition is not one‐size‐fits‐all. Rather, national government policies showed some potential for changing citizen health by modifying deep drivers that leverage the obesity syndemic (i.e. food supply chain, urban design and land use). Lowering the content of trans fatty acids and salt in food, taxes and subsidies, and banning food advertising contributed to promoting healthy diet behaviours to some extent (Table 4).^46^, ^47^, ^84^, ^85^, ^86^, ^87^, ^88^, ^89^, ^90^ However, as no single change in the behaviour created the obesity pandemic, no one policy can reverse it. We need to accumulate policies outlasting the usual political cycles to break vicious loops in governance, business, supply/demand and ecology.^91^ More than just relying on ‘willpower’, the engagement of citizens and communities is crucial for achieving these changes. To achieve this objective of engaging citizens and communities, clear narratives around body fat distribution and ‘lifestyle vital signs’ (i.e. waist circumference, cardiorespiratory fitness, food‐based overall diet quality and level of physical activity) should be developed. People can indeed profoundly influence obesogenic environments and food insecurity in their roles as voter/elected officials, employers, parents and customers. Their influence is directed to micro systems like family, worksites, schools, food retailers and their own communities daily, but citizens become decision‐makers of macro systems as well when they can vote for, advocate for and communicate their preferences with other decision‐makers about policies and actions. The European Commission showcased its commitment towards civilian efforts by launching the ‘HealthyLifestyle4All’ in 2022, a not funded support of voluntary pledges by State authorities, representatives of the sport movement and civil society organizations, cities and local governments. They are all expected to contribute to the goals of the HealthyLifestyle4All initiative, namely (i) increasing awareness of healthy lifestyle across all generations; (ii) favouring easier access to physical activity and healthy diets, with a special focus on inclusion; and (iii) collaborating for a holistic approach to well‐being. Similarly, the health policy advocacy organization ‘Trust for America's Health’ frames well the complexity and contentedness of obesity and recommend policy steps that include; (i) fully funding CDC's prevention programmes to reach every state, enhanced the access to nutrition support programmes; (ii) implementing mandatory front‐of‐package informative labelling on food packaging; (iii) increasing federal commitment to health and physical education and investing in active transportation projects (iv) closing tax loopholes and business‐cost deductions for advertising unhealthy food; and (v) increasing access to health insurance as for the ‘treat and reduce obesity act’.^92^ The European and US food policy frameworks provide a unique opportunity to examine large scale approaches to obesity prevention, while several non‐EU countries have successfully implemented innovative policies to combat diet‐related diseases. Mexico and Chile have emerged as global leaders in obesity prevention, enacting comprehensive food labelling systems, marketing restrictions and fiscal policies aimed at discouraging the consumption of ultra‐processed foods.^93^, ^94^ There, the purchases of sugary beverages and processed foods significantly declined through front‐of‐package warning labels and specific taxation, both approaches that remain highly debated within the EU/US due to industry pushback.^95^

**TABLE 4:** Summary of contemporary evidence about the effectiveness of policies fighting obesity worldwide.

Author	Year	Study design and time frame	Policy targets	Findings
Theis et al.^96^	2021	Mixed method for document review and analysis of UK government strategies 1992–2020	Diet *n* = 231 (33%) Physical activity *n* = 169 (25%) Non‐specific *n* = 289 (42%)	Policy proposals were mainly non‐interventionist. They relied on individual behaviour changes rather than shaping external influences
Itria et al.^97^	2021	Systematic review 2009–2019	16 worldwide studies on taxing SBB	Taxation on SSB results in decrease of OB prevalence, ranging from .99% to 2.7% points and greater extent in middle‐/high‐income countries. There was also a dose–response effect on SBB intake for taxes of 10% and 20%
Kwon et al.^84^	2021	Mixed method for online surveys and semi‐structured interviews 2016–2020	Impact of Food‐EPI initiative Government and non‐government Australian stakeholders	Food‐EPI Australia supported policy processes by enhancing the understanding of best practices in decision‐making and advocacy strategies
Griffin et al.^85^	2021	Analysis of policy discourses with a SDH perspective 2016–2019	‘Childhood Obesity: a plan for action’ UK programme	When considering social inequalities, the current approach highlights fundamental gaps between the proposed aims (healthy diets and opportunities for physical activity) and the need to frame them in the complexity and contestedness of OB
Rummo et al.^86^		Longitudinal study 2009–2016	Impact of the FRESH programme on 11,356 students (with a control group of 43,372 students) in New York city	The opening of FRESH supermarket providing access to affordable, healthy food options to low‐income neighbourhoods lead to a significant decrease in BMI among students who resided within .50 miles (vs. control group) in the 3–12 months of follow‐up period. The difference‐in‐differences (DiD) linear probability of changing BMI *z* score was (−.04 [95% CI −.06 to −.02]). A significant decrease in the likelihood of OB was also observed (DiD, −.01 [95% CI −.02 to −.002])
Kenney et al.^87^	2022	Cross‐sectional study with a mixed method 2019–2020	20‐min survey on the of implementation strategies to support HEPAST policies in US states childcare licensing providers	Given a substantial variability between states, the main challenges in HEPAST adoption were associated with financial constraints, staff turnover, and inadequate facilities. The implementation of federal food programmes was identified as the most critical aspect to address
Poitier et al.^88^	2022	This multi‐method case study 2019–2021	Document review of health policies combined with secondary semi‐structured interviews with stakeholders in the Caribbean region	National policies and community‐level interventions between 2008 and 2019 are thought to have contributed to reducing obesity prevalence from 49.2 to 43.7% between 2012 and 2019
Vega‐Salas et al.^89^	2022	Systematic review 2000–2021	Nine studies on school environmental interventions (i.e. nutritional and physical education) in Latin America and Caribbean	In two studies, interventions were effective in reducing the increase of BMI (mean difference of BMI‐for‐age‐percentile −.07 [95% CI −.12 to −.02]) and OB prevalence (mean difference in OB% .34 [95% CI .51–.91])
Rogers et al.^98^	2023	Two‐tier SDIL on drinks manufacturers to encourage reformulation of SSBs 2018–2019	UK children from state‐maintained English primary schools 4–5 years and 10–11 years old approximately 1 million	As compared to a counterfactual (adjusted for temporal variations in obesity prevalence) estimation, SDIL significantly reduced the OB prevalence ((defined as >95th centile on the UK90 growth charts)) in 10–11 years old children belonging to the most deprived quintiles (2.4% and 1.6% in girls and boys, respectively)
Economos et al.^99^	2023	Community coalition ‘Shape Up Under 5 Committee’, in Somerville, MA 2015–2017	Policy, systems and environment data were collected via the validated COMPACT Stakeholder‐driven Community Diffusion survey	Over 2 years, knowledge of (*p* < .001), and engagement with (*p* = .03), childhood obesity prevention increased significantly among the SUU5 Committee
Bijlani et al.^100^	2023	Go‐Golborne intervention Shape the local environment across multiple settings with a joint approach engaging many local government and community stakeholders 2016–2019	UK children from six local schools aged 6–11 years 1650	After 3 years of follow‐up, there were reductions in SSB, and fruit/vegetable consumption (adj *β* −.43 occasions/day, adj *β* −.22 portions, respectively) and car travel to and from school as well (adj OR .19 [95% CI .06–.66]), while screen time increased (high vs. moderate/low: OR 2.30 [95% CI 1.36–3.90])
Kenney et al.^90^	2024	Cost‐effectiveness analysis 2010–2019	Effect of food package change on obesity risk US children 2–4 years old included in the WIC	WIC is estimated to have reached 14 million US children in the period from 2010 to 2019. As a result, approximately 62,700 cases of childhood obesity were prevented, all within households with low incomes. This improvement in health equality led to an estimated gain of $10,600 per quality‐adjusted‐life year

*Note*: The search for studies was conducted up to March 2024 in the PubMed database. The terms identified for the PubMed search were (obesity [Title]) AND (policy [Title/Abstract]) AND ((sugar‐sweetened beverages [Title]) OR (tax [Title]) OR (government [Title]) OR (initiative [Title])) OR (stakeholder [Title]) from 2021. The same terms were also used to identify systematic reviews and meta‐analysis in the period from 2021 The search was limited to articles that were written in English and were published from 2023. The database searches were complemented with manual review of the reference lists of relevant articles. Finally, the search results were further refined by including studies that effectively provided time‐trend analyses.

Abbreviations: BMI, body mass index; FRESH, Food Retail Expansion to Support Health; HEPAST, healthy eating, physical activity, and screen time; OB, obesity Food‐EPI: Healthy Food Environment Policy Index; SBB, sugar‐sweetened beverages; SDH, social determinants of health; SDIL, soft drinks industry levy; WIC, Special Supplemental Nutrition Program for Women, Infants, and Children.

The lesson of the COVID‐19 pandemic has further highlighted how frail the food security is in the EU‐27 countries.^14^ While the EU food trade sector demonstrated greater resilience compared to other industries, the temporary decline in imports threatened food access in several member states, particularly in net food‐importing countries such as Malta, Cyprus, Croatia, Greece, Slovenia and Portugal. From the perspective of economic access, rising food prices contributed to a higher percentage of the population reporting that they could not afford a meal with meat, fish or a vegetarian equivalent every 2 days. This percentage increased to 8.1% in 2020, compared to 6.8% in 2019 and despite a slight reduction in 2021 (7.3%), it remained above pre‐pandemic levels. Additionally, the FAO index of severe food insecurity showed a deterioration in 18 EU countries between 2019 and 2021, indicating that the health crisis exacerbated existing vulnerabilities despite national and EU‐level economic support measures. These findings reinforce the urgency of incorporating food security into obesity prevention policies. Addressing economic insecurity alone is insufficient and governments must also tackle systemic challenges in food supply chains, the affordability of fresh and nutritious food and regional disparities in food access. In particular, the pandemic experience suggests that food security can no longer be considered a marginal or temporary issue but rather a strategic priority for economic and social resilience in European public health policies. Integrating food security into public health and obesity prevention strategies is not just a matter of economic resilience but essential to ensure equitable and sustainable access to food for the entire European population, particularly in anticipation of future global crises.

Finally, we must consider the double‐edge sword of social media. The COVID‐19 pandemic deeply amplified and modified its usage, resulting in a massive increase of obesity‐related posts and interactions during the first year of the pandemic. However, increased coverage of obesity by mainstream media both raised awareness and fuelled weight‐related stigma. While obesity‐related health organizations and weight loss applications do not sufficiently emphasize the burden of obesity on their social media platforms, the clinical and commercial content of which has dubious accuracy and may easily fool patients and the general population.^101^ Specific algorithms can be leveraged to rapidly identify deleterious or fake content with minimal supervision^102^ but these cannot replace a commitment of healthcare providers to harness the power of technologies for addressing challenges in obesity medicine.

## 4 TASK 1. POINT TO POINT SUMMARY

**Table:** 

Knowledge gaps	Long‐range robust longitudinal data on obesity transition are mostly lacking, as most of the studies are meta‐analyses collected across decades Geographical and socio‐economic disparities in obesity transition Migration and food security as emerging drivers of obesity Limited use of AI/ML in obesity transition research Estimated temporal trends are also arbitrarily categorized across decades without clear rationale Most studies define overweight/obesity according to BMI values only Even the BMI cut‐offs may not be well justified
Implementation strategies	Establish standardized cohort studies and harmonized data collection to track long‐term obesity trends at global, national and subnational levels Develop composite indices integrating BMI, waist circumference, body composition and metabolic biomarkers to more accurately classify obesity transition stages Incorporate subnational socio‐economic and environmental data to capture heterogeneity within countries and assess urban–rural differences Investigate the impact of migration and food security by integrating multi‐dimensional food security indicators and analysing dietary adaptation among immigrants Apply AI and machine learning to predict obesity trends using big data from wearable devices, electronic health records and urban planning databases. Break the deep drivers that leverage the obesity syndemic Consider individual, local, community, country‐level and global initiatives Collect unbiased data on programme success and avoid over‐confident beliefs that well‐intentioned interventions would work simply because they are well‐intentioned Consider interventions by citizens at different roles (e.g. voter, employers, parents, customers, scientist) Exploit new technological opportunities, such as artificial intelligence
Potential issues	Data fragmentation across different regions and lack of standardized methodologies limit comparability and long‐term trend analysis BMI‐based classification overlooks body composition and metabolic health, leading to misclassification and ineffective public health measures. Economic and policy variations make it difficult to apply uniform strategies; urban–rural disparities in data collection affect accuracy. Food security is often studied in economic terms, neglecting cultural, geographic, and accessibility barriers; migrant dietary behaviours remain underexplored. AI requires high‐quality, real‐time data; ethical concerns about data privacy and bias in predictive models need to be addressed Detrimental use of social media with misguided commercial content and fake news Low propensity of healthcare providers to harness social media
Potential benefits	Improved prediction of obesity trends, better policy planning and targeted interventions to prevent rapid obesity increases More precise identification of at‐risk populations, enabling better‐tailored interventions and classification of obesity phenotypes Better understanding of how socio‐economic and environmental factors drive obesity disparities, leading to localized intervention strategies Enhanced knowledge of how acculturation, food insecurity and economic instability influence obesity, informing targeted policies for migrant populations AI‐driven models will refine obesity surveillance, provide real‐time insights and enable precision public health interventions.

## 5 TASK 2. FROM OBESITY TO OBESITIES—THE GAPS IN CONSIDERING ITS PHENOTYPIC HETEROGENEITY

### 5.1 Background

While BMI is still considered the primary metric for categorizing overweight/obesity at a population level, it represents a crude measure of excess adiposity, unable to recognize individual differences in regional body fat distribution and reflects both fat and lean tissue mass, without differentiating between fat and muscle as the source(s) of excess body mass. Instead, the contemporary challenge of tackling high CV risk phenotypes of obesity through a combination of personalized approaches and population‐based solutions calls for a deeper—still unmet—awareness of its wide heterogeneity, now commonly referred to as ‘obesities’.

The first differentiation between ‘android’ and ‘gynoid’ types of obesity dates back to 1956 and marked the starting point in the quest for extra body weight categorization.^103^ Countless attempts have been made to define phenotypes of body fat distribution through anthropometric and metabolic measures. One of the rationales for defining obesity is to provide a measure of risk continuum for cardiometabolic disease, through its association with cardiometabolic risk clusters.^104^ While Prof. Reaven looked at insulin resistance as a key central tenet for metabolic abnormalities^105^ and the NIH established thresholds values for waist circumference,^106^ the National Cholesterol Education Panel Adult Treatment Panel III (NCEP ATP III) firstly proposed simple tools for clustering individuals likely to be characterized by abdominal obesity and insulin resistance and referred to as having metabolic syndrome (MetS).^107^ Even though Prof. Reaven himself finally questioned the utility of diagnosing MetS,^108^ the subsequent timeline witnessed multiple risk factors and variables added to what has been recently named the ‘MetS reloaded’.^109^ Similarly, several obesity stakeholders still get stuck in the recommendation of BMI alone for obesity definition and risk stratification despite being intrinsically limited and a harbinger of paradoxes.^110^

### 5.2 Summary of the evidence

**FIGURE 1:**
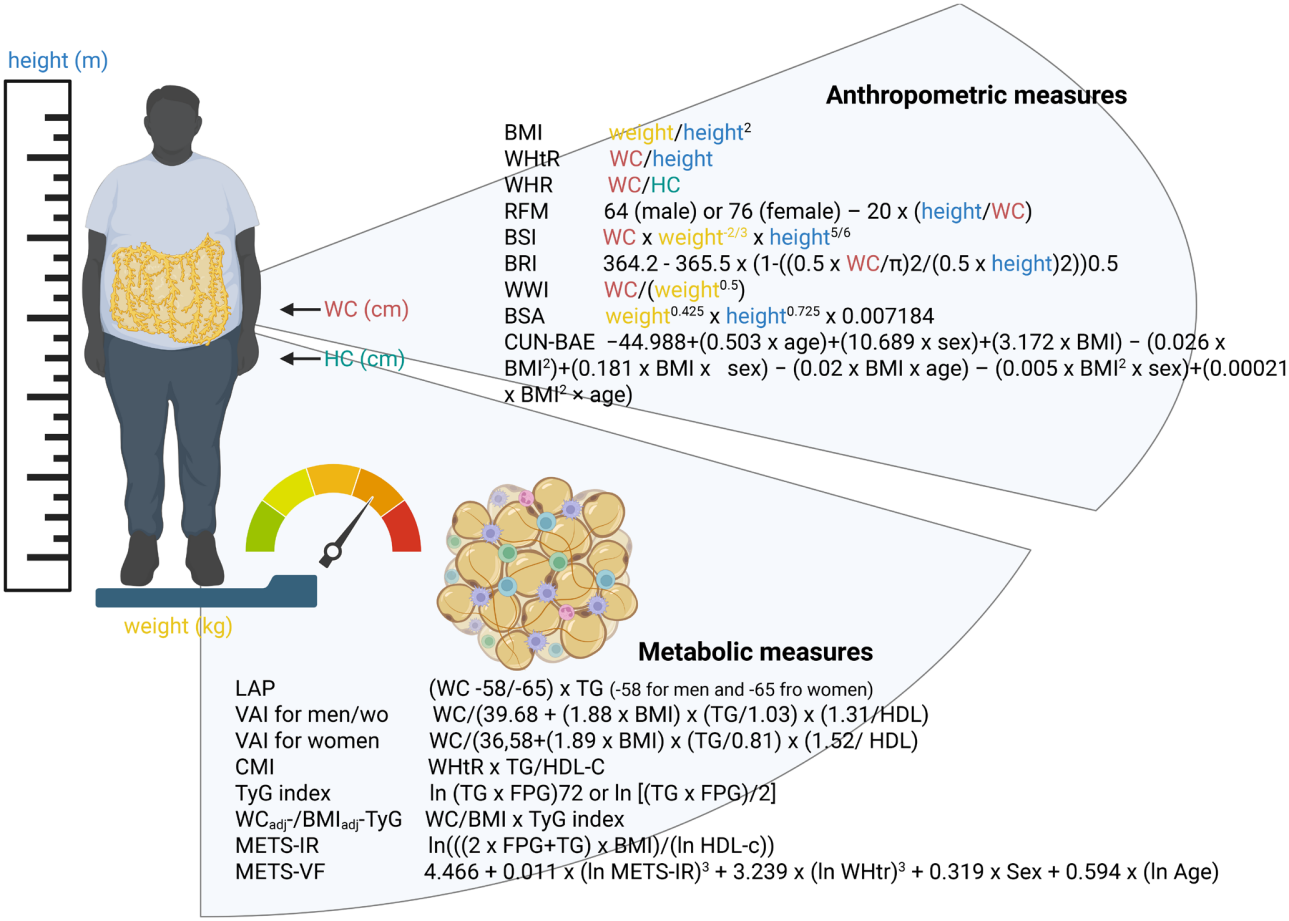
Summary of contemporary methods for obesity assessment/phenotyping. Based on anthropometric and biochemical parameters there are a plethora of proposed criteria for obesity assessment and phenotyping. BMI, body mass index; BRI, body roundness index; BSA, body surface area; BSI, body shape index; CMI, cardiometabolic index; CUN‐BAE, Clínica Universidad de Navarra‐Body Adiposity Estimator; LAP, lipid accumulation product; METS‐IR, metabolic score for insulin resistance; METS‐VF, metabolic score for visceral fat; RFM, relative fat mass; TyG, triglyceride‐glucose index; VAI, visceral adiposity index; WHR, waist circumference‐to‐hip circumference ratio; WHtR, waist circumference‐to height ratio; WWI, weight‐adjusted waist index. Created by Biorender.com.

As early as 2011, the Emerging Risk Factor Collaboration reported how BMI, waist circumference (WC) and waist‐to‐hip ratio (WHR)—either alone or in combination—do not considerably improve estimation of cardiovascular (CV) disease risk when additional information (i.e. blood pressure, cholesterol and glucose levels) is available.^111^ In other two studies at least, the excess risk of adverse CV events was mostly explained by the coexistence of traditional CV risk factors alone (66%–69%).^112^, ^113^ Furthermore, several meta‐analyses testing the predictive role of BMI towards a poor CV outcome have generally proved to be inconclusive, reporting a U‐shape correlation.^114^, ^115^ Especially when defined based on BMI alone, obesity emerges as a markedly heterogeneous condition. Notably, WC considerably varies for any given BMI and across BMI categories, with an elevated WC being associated with a greater burden of cardiometabolic disturbances and with an increased mortality risk in every BMI category. Although thresholds of WC within BMI categories were developed,^116^ the conversion from continuous to categorical data results in the loss of important information, ultimately reducing prediction performance. The strength of the association between WC and CV risk would then not fully realize until adjustment for BMI,^117^ as argued in a seminal joint consensus statement from the working groups of International Atherosclerosis Society and International Chair on Cardiometabolic Risk.^118^ However, a plethora of anthropometric measures—treated as continuous variables—are candidate to implement the discrimination of prevalent and incident unhealthy phenotype (Figure 1).^119^, ^120^, ^121^, ^122^, ^123^, ^124^, ^125^, ^126^, ^127^, ^128^, ^129^, ^130^, ^131^, ^132^, ^133^, ^134^, ^135^, ^136^, ^137^, ^138^, ^139^, ^140^, ^141^, ^142^

### 5.3 Knowledge gap

Upon closer examination, this roundabout on anthropometric measures further accounts for the wide heterogeneity in obesity phenotypes. Accurately predicting CV risk in people living with obesity (PlwO) is challenging when limited to classic risk factors. Prediction models like the Pooled Cohort Equation and SCORE never included BMI or WC as a biomarker but exhibit only moderate performance in subjects with obesity and/or with type 2 diabetes (T2D), even when classical risk factors are homogeneously distributed.^143^, ^144^ In the same way, the severity of T2D risk factor is conditioned by bodyweight status, whereas the association between anthropometric measure and CV risk is markedly different across age strata.^111^, ^145^ While adapting risk prediction models to specific subgroups did not yield improved performance, increasing their complexity might be optimal but not attractive. Accounting for BMI categories, the relationship with health outcomes is further complicated by the concept of obesity paradox, characterized by a lower likelihood of an adverse prognosis in individuals with established CVDs and overweight/mild obesity compared with those of normal or underweight. An obesity paradox has been reported for both CV and non‐CV disease/mortality in people with overweight and mild obesity, and has often been explained to be, at least in part, related to better cardiorespiratory fitness (the so‐called ‘fat but fit’ phenotype) and the metabolically healthy obesity (MHO) phenotype. Especially the latter stands at the centre of the problem due to the lack of a universally accepted definition. More than 30 different definitions of MHO have been used in the literature, all while still allowing the presence of cardiometabolic risk factors—usually two or fewer defining criteria for metabolic syndrome.^146^ Approximately 10% of patients deemed to have MHO exhibit no tenets of metabolic syndrome and an even lower figure (5%) is estimated for those having normal insulin sensitivity at HOMA‐IR as well. Individuals classified as MHO but stable over time and without any metabolic abnormality are argued to share the same CV risk profile as the metabolically healthy lean phenotype. This critical yet poorly addressed issue found some support in the European Prospective Investigation into Cancer and Nutrition Study, where the relative risk of overweight/obesity was similar to that of lean individuals when accounting for no metabolic abnormalities.^147^ With the same limitations, the co‐existence of metabolic abnormalities is currently used to discriminate the clusters of metabolically healthy/unhealthy normal weight (MHNW/MUNW) and metabolically unhealthy obesity (MUO).^148^ Once again, a crude categorization into a few risk groups results in the loss of important information as the CV risk stratification related to metabolic abnormalities deals with qualitative, and temporal aspects as well. The distribution of CV risk is generally higher, but the discriminative performance of prediction models is lower when insulin resistance/diabetes is present.^149^ However, the average effect size estimated by subgroup‐specific prediction models indicates only moderate performance, thus suggesting further layers of complexity. Female sex and healthier behaviours are common and intriguingly found in MHO. Beside the higher adherence to healthy dietary pattern (e.g. Mediterranean diet) irrespective of total energy intake,^150^ people with MHO also more likely to have better cardiorespiratory fitness (Table 5).^151^, ^152^, ^153^, ^154^, ^155^, ^156^, ^157^, ^158^, ^159^, ^160^, ^161^, ^162^, ^163^, ^164^, ^165^, ^166^, ^167^ Ultimately, whether MHO really means ‘disease‐free’ may only be related to a single point in time. Despite lower than MUO phenotype, CV risk in MHO—assuming this classification holds true—is substantially higher than in lean individuals, rising to about 45% as the observation period lengthens.^168^ Moreover, MHO is increasingly considered a transition stage to MUO associated with a further increase in CV risk (Table 6).^169^, ^170^, ^171^, ^172^, ^173^, ^174^, ^175^, ^176^, ^177^

**TABLE 5:** Summary of the evidence on the ‘fatness but fitness paradox’.

Author	Year	Study design and time frame	Population characteristics	Findings
Barry et al.^151^	2018	Meta‐analysis 8 studies 1989–2017	General population characterized for BMI and CRF	People with OW but not OB‐fit had a significant (25%) increased of mortality risk compared with compared to lean‐fit individuals
Ortega et al.^152^	2018	Meta‐analysis 67 + 11 studies 2001–2018	MHO and MHNW (*n* = 69,995)	People with MHO spend less time in sedentary behaviour MHO have a borderline significant higher risk of all‐cause mortality and CV mortality/morbidity compared to MHNW individuals when adjusted for physical activity (33% and 24%, respectively)
Qiu et al.^153^	2019	Meta‐analysis 15 studies Up to 2018	General population (*n* = 57, 278) statin users (12,311) characterized for BMI and CRF	For every 1‐metabolic equivalent increase in CRF there was a significant 10% reduction in risk of developing T2D. Risk reduction in OW/OB was greater in fit than unfit categories as compared with lean individuals
Lee et al.^154^	2020	Meta‐analysis 8 studies 1990–2019	General population (*n* = 5474) characterized for BMI and CRF	Risk of MetS increased with low CRF (RR 3.6 [3.1–4.2]) and OB (RR 1.6 [1.3–2.0]). CRF (≥8.39 metabolic equivalent) decreased by 77% the risk of MetS independently of OB status (RR .23 [.1–.4])
Cristi‐Montero et al.^155^	2021	Cross‐sectional	European adolescents (*n* = 525) 12–17 years old from the Healthy Lifestyle in Europe by Nutrition in Adolescence study	CRF plays an important role in mediating the association between fatness and CMR: BMI (*β* = .06), waist‐to‐height ratio (*β* = 4.28), fat mass index (*β* = .06); *p* < .01 for all
Santaliestra‐Pasías et al.^156^	2021	Longitudinal study 2007–2010	European adolescents (*n* = 289) 6–9 years old From the IDEFICS study	Training muscular fitness during childhood and reach top CRF were associated with an 82% and 77%, independently of sociodemographic and lifestyle indicators
Leonard et al.^157^	2021	Longitudinal study 1979–2019	US general population (*n* = 3534) 21–89 years from the Cooper Center Longitudinal Study	A 1‐MET increase in fitness reduced the odds for MetS by 23% (OR .77 [95% CI .70–.84]), while adjusting for body mass changes up to 10 years
Navarrete‐Villanueva et al.^158^, ^159^	2021	Longitudinal study 2008–2018	Spanish elderly (*n* = 2299) Older 65 years Spanish EXERNET‐Elder project	Using the low fat‐fit pattern as the reference, significantly increased mortality was noted in High Fat‐Unfit (HR: 1.68; [95% CI 1.06–2.66]) and low fat‐unfit (HR: 2.01, CI: 1.28–3.16) groups. In a subgroup of 1709 elderly, the high‐Fat‐Unfit pattern retained the most significant drug consumption rate and had the highest percentage of poly‐medicated subjects
Weisstaub et al.^160^	2022	Cross sectional	Chilean school children (*n* = 452) 7–9 years old CRF and MF	Fitness was associated with lower metabolic risk (*p* for trend <.001). The odds were lower in lean children (OR .08 [.04–.16]) and those with excess weight but high physical fitness (OR .43 [.23–.78])
Kondakis et al.^161^	2022	RCT	European adolescent (*n* = 1053) 13–18 years old VO_2_max (20 m multi‐stage fitness test)	VO_2_max is an independent predictor of high HOMA‐IR alongside with BMI in male and FMI/TV watching in females (*p* > .01 for all)
Makituomas et al.^162^	2022	Cross‐sectional	European adolescent involved in competitive sports vs. school/non‐competitive sport sample (*n* = 475/936) 14–16 years old	Adolescent involved in competitive sports perceived their body size as about the right size more frequently than reference group with some sex‐related difference (68% vs. 47% in girls; 74% vs. 61% in boys)
Wing et al.^163^	2022	Randomized interventional trial	US sedentary adults (*n* = 607) 65–84 years old self‐reported cognitive complaints, but without a clinically defined dementia	VAT (*r* = .149), TST (*r* = .087), and maximal aerobic capacity (*r* = −.088) are all linearly associated with brain‐predicted age at MRI
Navarro‐Lomas et al.^164^	2023	Cross‐sectional	Healthy and sedentary European adults (*n* = 150) 18–65 years old	FFIs were calculated as the quotient between CRF and fatness markers: FFI_WHR_, FFI_FM%_, and VAT (FFI_VAT_). All indexes—but mainly FFI_VAT—_were associated with heart rate variability as surrogate index of cardiometabolic health and risk
Jackson et al.^165^	2024	Cross‐sectional	OW/OB adults enrolled in a behavioural weight loss programme including MVPA (*n* = 375) 18–55 years old	Some people with OW/OB may be more active than they perceived themselves to be. MVPA ≥150 min/week may have favourable effects on weight and adiposity status
Brand et al.^166^	2024	Cross sectional	Children and adolescents (*n* = 2786) aged 6–17 years	Significant interactions were found between physical activity and the unfit/fat group (*β* = −.001; *p* = .001) in adolescents. Physical activity for 330 minutes per week reduced the number of MetS criteria

*Note*: The search for studies was conducted from 2021 up to March 2024 in the PubMed database (from 2018 for meta‐analyses). The terms identified for the PubMed search were ((obesity) OR (overweight)) AND ((fitness [title])) AND (cardiometabolic [title]). The same terms were also used to identify systematic reviews and meta‐analysis in the same period. The search was limited to articles that were written in English. The database searches were complemented with manual review of the reference lists of relevant articles. Finally, the search results were further refined by including studies that effectively provided time trend analyses.

Abbreviations: BMI, body mass index; CMR, cardiometabolic risk; CRF, cardiorespiratory fitness; CV, cardiovascular; FFI, fit‐fat index; FM, fat mass; FMI, fat mass index; MetS, metabolic syndrome; MF, muscular fitness; MHNW, metabolically healthy normal weight; MHO, metabolically healthy obesity; MRI, magnetic resonance imaging; MVPA, moderate‐to‐vigorous physical activity; OB, obesity; OR, odds ratio; OW, overweight; RR, relative risk; T2D, type 2 diabetes; TST, total sleep time; VAT, visceral adipose tissue; VO_2_max, maximum rate of oxygen consumption attainable during physical exertion; WHR, waist‐to‐height ratio.

**TABLE 6:** Summary of the contemporary evidence on transition from healthy to unhealthy obesity phenotype.

Author	Year	Study design and time frame	Population characteristics	Findings
Abiri et al.^169^	2022	Meta‐analysis of 7 studies 2018–2020	MHO (*n* = 7,720,165) Mean follow‐up 11 years	Transitional phenotype has a greater CVD risk (HR = 1.4 [1.2–1.6]). The unstable phenotype was similarly associated with CVD risk in both sex
Zhang et al.^170^	2023	Longitudinal study	Apparently healthy Chinese subjects (*n* = 17,309) Mean age 63 years Median follow‐up 9.9 years	MHO was the most unstable phenotype with a transition rate of 53.9%. While the MHO prevalence at baseline was the lowest (3.6%), 31.8% of those in MHO progressed to MUO in ~1 year. The transition intensity from MHO to MUO (.56) was the second highest after that from MUNW to MHNW (.64)
Wei et al.^171^	2023	Longitudinal analysis from the Dongfeng‐Tongji Cohort Study	Retired Chinese employers (*n* = 9742) 20–60 years old Mean follow‐up 6 years	The risk of developing T2D was higher in stable MHOO vs. MHNW (HR 1.8 [1.3–2.5]) and transition from MHOO to both MUNW (3.0 [1.8–4.9]) and MUOO phenotypes (HR 3.4 [2.5–4.5])
Xin et al.^172^	2023	Longitudinal study 2010–2015	Chinese community residents (*n* = 6260) >40 years old Median follow‐up 4.3 years	The risk of developing subclinical atherosclerosis was related to MU status independently of fatty liver content (HR 1.7 [1.3–2.1] and 2.6 [1.9–3.5]). Fatty liver rather conditioned a persistent or progression to MU phenotype (3.1 [1.2–7.9] and 4.9 [3.3–7.3], respectively) with lower rate of regression
Kouvari et al.^173^	2023	Longitudinal analysis of Framingham Offspring Study 1999–2014	US community residents (*n* = 2892) Mean age 61 years Median follow‐up 13 years	MHNW and MHO did not differ in cognitive function/decline over time. Only a lower processing speed/executive functioning scale score was recorded in MHO participants with 1 or 2 NCEP ATP III criteria (*β* = −.76; *p* = .030)
Zhang et al.^174^	2023	Meta‐analysis 17 studies Until 2022	Worldwide subjects (*n* = 8,270,923) Age range 43–61 follow‐up range 4–24 years	Transition to MH status significantly lowered the risk in both people with NW and OW/OB. In persistent MU status the progression from NW to OW/OB increase CV risk but not the incidence of T2D
Zhao et al.^175^	2023	Longitudinal study 2010–2022	Chinese community without CVD (*n* = 54,441) Age classes (<55, 55–65, 65–75, and ≥ 75 y) Median follow‐up 10 years	MUO at baseline led a greater CVD risk, which was higher in <55 years group (HR 2.7 [2.0–3.6]) than and lower in ≥75 (HR 1.5 [1.1–2.1]). CVD risk was still increased in both baseline and persistent MHO status but lowered across age groups
Xiao et al.^176^	2023	Randomized interventional trial	Metabolically healthy Chinese children (*n* = 6424) 6–16 years old 2 years follow‐up	The study linked the transition into MUO with vitamin D deficiency. Lower levels of vitamin D were also linked to a lower rate of regression to MHNW
Chia et al.^177^	2023	Longitudinal analysis of the GUSTO study 2009–2010	Mother‐offspring cohort (*n* = 546) Followed‐up from the age of 2–8 years	Once categorized for lifestyle patterns the unhealthy one was associated with increased odds of metabolic syndrome score (*β* = .85 [.20–1.49]) but not BMI or other anthropometric parameters

*Note*: The search for studies was conducted from 2023 up to March 2024 in the PubMed database (from 2022 for meta‐analyses). The terms identified for the PubMed search were (((((body mass index) OR (obesity)) OR (overweight [title])) AND (transition) [title])). The same terms were also used to identify systematic reviews and meta‐analysis in the same period. The search was limited to articles that were written in English. The database searches were complemented with manual review of the reference lists of relevant articles. Finally, the search results were further refined by including studies that effectively provided time‐trend analyses.

Abbreviations: BMI, body mass index; CVD, cardiovascular disease; HR, hazard ratio; MHNW, metabolically healthy normal weight; MHO, metabolically healthy obesity; MHOO, metabolically healthy overweight/obesity; MUNW, metabolically unhealthy normal weight; MUO, metabolically unhealthy obesity; MUOO, metabolically unhealthy overweight/obesity; OB, obesity; OW, overweight; T2D, type 2 diabetes.

The intrinsic limitation of BMI, the potential for implementing through WC and the attempt to combine metabolic measures all emphasize the gaps in dealing with the heterogeneity of obesity. Taking for granted the concept of adiposopathy and its central tenets (i.e. shift towards visceral and ectopic fat deposition, insulin resistance and inflammation), the pathogenic potential of excess body fat is deeply conditioned on adipose tissue dysfunction and ectopic deposition rather than on increased of fat mass alone.^178^ In other words, body fat quality and location matter. Chronic low‐grade inflammation is an established upstream mediator of high‐risk obesity phenotypes,^179^, ^180^ strongly dependent on visceral adipose tissue as source and driver.^181^, ^182^ Furthermore, it also seems to predict the benefit from interventions: the higher at baseline the better the metabolic improvement.^183^, ^184^ Similarly, combining anthropometric and biochemical biomarkers of metabolic dysfunction may aid in obesity phenotyping and many efforts in the last decades have focused on this topic (Figure 1).^119^, ^120^, ^121^, ^122^, ^123^, ^124^, ^125^, ^126^, ^127^, ^128^, ^129^, ^130^, ^131^, ^132^, ^133^, ^134^, ^135^, ^136^, ^137^, ^138^, ^139^, ^140^, ^141^, ^142^, ^185^, ^186^, ^187^, ^188^, ^189^, ^190^, ^191^, ^192^, ^193^, ^194^, ^195^, ^196^, ^197^, ^198^, ^199^, ^200^, ^201^, ^202^, ^203^, ^204^, ^205^, ^206^, ^207^, ^208^, ^209^, ^210^, ^211^, ^212^ The subsequent Task will delve deeper into how fat distribution differentially impact of CV disease presentation and outcomes.

Adding a further layer of complexity, the European Society for Clinical Nutrition and Metabolism and the European Association for the Study of Obesity are upfront in reaching a consensus on definition and diagnosis for sarcopenic obesity (SO), standing for obesity accompanied by reduced muscle function and mass, later extended to dynapenic obesity (DO), standing for obesity accompanied by reduced muscle strength.^213^, ^214^, ^215^ The loss of skeletal muscle mass and function would result in a bidirectional pathogenic interaction with body fat mass leading to a synergistically higher metabolic risk. The groups advocate for considering SO/DO as a single entity but characterized by two distinct phenotypic traits. They launched the Sarcopenic Obesity Global Leadership Initiative to test the validity of a proposed diagnostic algorithm based on normalization of skeletal muscle mass/function to body mass, aiming at identifying rather than defining ‘relative or inadequate muscle mass’. Clinical data are indeed still insufficient to support an integrated index for SO definition.^136^, ^216^, ^217^, ^218^, ^219^, ^220^

### 5.4 Implementation strategies and potential issues

An alternative approach to a priori definitions of metabolic health is to empirically derive a new definition exploring risk factors for mortality among PlwO. Applied to a representative sample from NHANES‐III of 12,341 individuals and externally validated in an independent UK Biobank cohort, MHO was defined by a systolic blood pressure < 130 mm Hg without any medication, waist‐to‐hip ratio less than .95 for women and less than 1.03 for men, and no diabetes.^221^ This metabolic profile would be characterized by no excess CV risk as compared with MHNW individuals, but opposite results have also been reported.^222^

In any case, the clinical relevance of cluster analyses for cardiometabolic risk stratification is forthcoming.^223^, ^224^, ^225^, ^226^ While important variables were not routinely available in clinical settings, digital phenotyping of obesity is rapidly gaining implementation. The advance in artificial intelligence (AI) allows for the handling of multimodal datasets, encompassing not only quantitative but also qualitative and temporal changes in metabolic health. Serial measures are now easy to collect with consumer wearable devices in free‐living states and are expected to critically contribute to characterize longitudinal change in phenotype/cluster assignment and overcome current criticisms in recognizing disease progression.^227^

However, the question arises of whether to identify further sub‐phenotypes of obesity or to better characterize the well‐established ones. Many efforts in the last decades have focused on metabolic phenotyping to implement the paradigm of adiposopathy (Figure 1).^119^, ^120^, ^121^, ^122^, ^123^, ^124^, ^125^, ^126^, ^127^, ^128^, ^129^, ^130^, ^131^, ^132^, ^133^, ^134^, ^135^, ^136^, ^137^, ^138^, ^139^, ^140^, ^141^, ^142^, ^185^, ^186^, ^187^, ^188^, ^189^, ^190^, ^191^, ^192^, ^193^, ^194^, ^195^, ^196^, ^197^, ^198^, ^199^, ^200^, ^201^, ^202^, ^203^, ^204^, ^205^, ^206^, ^207^, ^208^, ^209^, ^210^, ^211^, ^212^ Notably, machine learning algorithms are also unveiling metabolomics signatures of obesity—also referred to as metabolic BMI (mBMI) score—and their differences across phenotypes/clusters.^228^, ^229^ The creation of a lipidome‐based BMI score has been shown upfront for improving patient stratification in a train/test on two large Australian cohorts. Although not yet ready for replacing existing risk scores for cardiometabolic diseases, mBMI may track the hidden residual risk associated with both prevalent and incident CV risk.^230^ The mBMI improved with the intake of fruits and fibre or physical activity but increased with prolonged TV viewing time. Those appear as valuable insights into an individual's metabolic risk profile, enabling targeted interventions to address specific metabolic health concerns prior to the onset of disease.^231^, ^232^, ^233^ Machine learning approaches may also maximize the efforts in discovering specific features of sarcopenic obesity^234^, ^235^ or even predict them.^236^ Finally, whether a panel integrating classical markers, multiomics, lifestyle and behavioural patterns, psychological traits and additional host factors like gut microbiota may impact obesity prevention is a further scoping effort to which AI and machine learning are called upon to respond.^237^, ^238^ More specifically, keeping/developing a healthy adiposity phenotype would represent a critical step forward to intercept people prone to develop metabolically unhealthy signatures.

Nevertheless, additional paradoxes emerge on the obesity horizon. While unhealthy forms of obesity are widely recognized to accelerate the pace of ageing,^239^, ^240^, ^241^ there seems to be some protective effect of high BMI on survival that gradually increases with age.^242^ Clonal haematopoiesis of indeterminate potential (CHIP) somewhat explains of these controversies. While CHIP is upfront—albeit debated^243^, ^244^, ^245^—in cardiovascular risk stratification as a hallmark of ageing‐related inflammation,^246^ such an acquisition of somatic mutations is associated with obesity in mice and with an increase in the waist‐to‐hip ratio in human beings.^243^ Nevertheless, whether clonal haematopoiesis driven mutations would predispose PlwO to metabolic disturbances and CV risk remains controversial.^245^

The transforming potential of AI is destined to rethink the concept of BMI and obesity, as well as their management. AI‐ready data may be collected from metabolomics and accelerometry but further efforts in standardizing big data and establishing causal inference are needed.^237^ Implementing AI and machine learning techniques are critical steps that still require time, effort and human supervision. Meanwhile, obesity‐devoted scientists and clinicians are tasked with spreading awareness on how the strength of the association between WC and CV risk is fully realized only when adjusted for BMI. This appears to be the contemporary and preferable tool to categorize and stratify patients as well as the desirable method to design/analyse clinical studies.

## 6 TASK 2. POINT TO POINT SUMMARY

**Table:** 

Knowledge gaps	Implementing BMI with specific waist cut‐off remains challenging, despite its intrinsic limitations and often being a harbinger of paradoxes The transition across obesity phenotypes is poorly addressed in clinical settings The role of lifestyle factors in obesity phenotype characterization and transition is lacking There is no consensus definition and diagnostic algorithm for MHO and sarcopenic/dynapenic obesity
Implementation strategies	Spreading awareness on how the strength of the association between WC and CV risk is fully realized only when adjusted for BMI Standardizing and harmonizing protocols for WC measure Validating other anthropometric measures need to be validated and eventually implemented to BMI and WC Considering, sex‐ and ethnicity‐specific cut‐off points need to be considered for anthropometric measurements and obesity phenotyping Testing the use of continuous vs. categorical variables as a strategy to implement obesity phenotyping and risk prediction Validating the use of metabolic scores Gathering knowledge on omics and unveiling the role of ageing process in obesity biology to foster an accurate phenotyping Exploiting advantages in the field of AI to promote a comprehensive approach to obesity that encompass quantitative measurements, qualitative assessment, over time modification in obesity phenotype and lifestyle factors
Potential issues	Development of a comprehensive multi‐omics phenotyping could require a wide multidisciplinary team Challenging collective ability to implement even simple and intuitive procedure for diagnosis and stratification of obesity into clinical practice

## 7 TASK 3. OBESITY AND CARDIOVASCULAR DISEASE: GAP IN STUDY DESIGN AND PATIENT ASSESSMENT

### 7.1 Background

The estimated risk of death associated with obesity increases by 30% for each 5 kg/m^2^ increase in BMI in overweight individuals, with most deaths due to CV disease.^247^, ^248^ However, CV risk may considerably vary for a given BMI at individual level. While obesity is a risk factor for many diseases, cardiometabolic afflictions account for most of the disease burden in this class of patients.^247^, ^249^ The strength of the association between obesity and cardiometabolic conditions increases exponentially among obesity classes, with individuals classified as severely or morbidly obese (BMI ≥ 40) showing a 15‐fold higher risk of developing T2D and overt atherosclerosis including myocardial infarction and ischaemic stroke.^250^, ^251^ In addition to the classic association with atherosclerotic afflictions, obesity also enhances the risk of heart failure with preserved ejection fraction (HFpEF), atrial fibrillation, sudden cardiac death and valvular defects.^252^, ^253^

**FIGURE 2:**
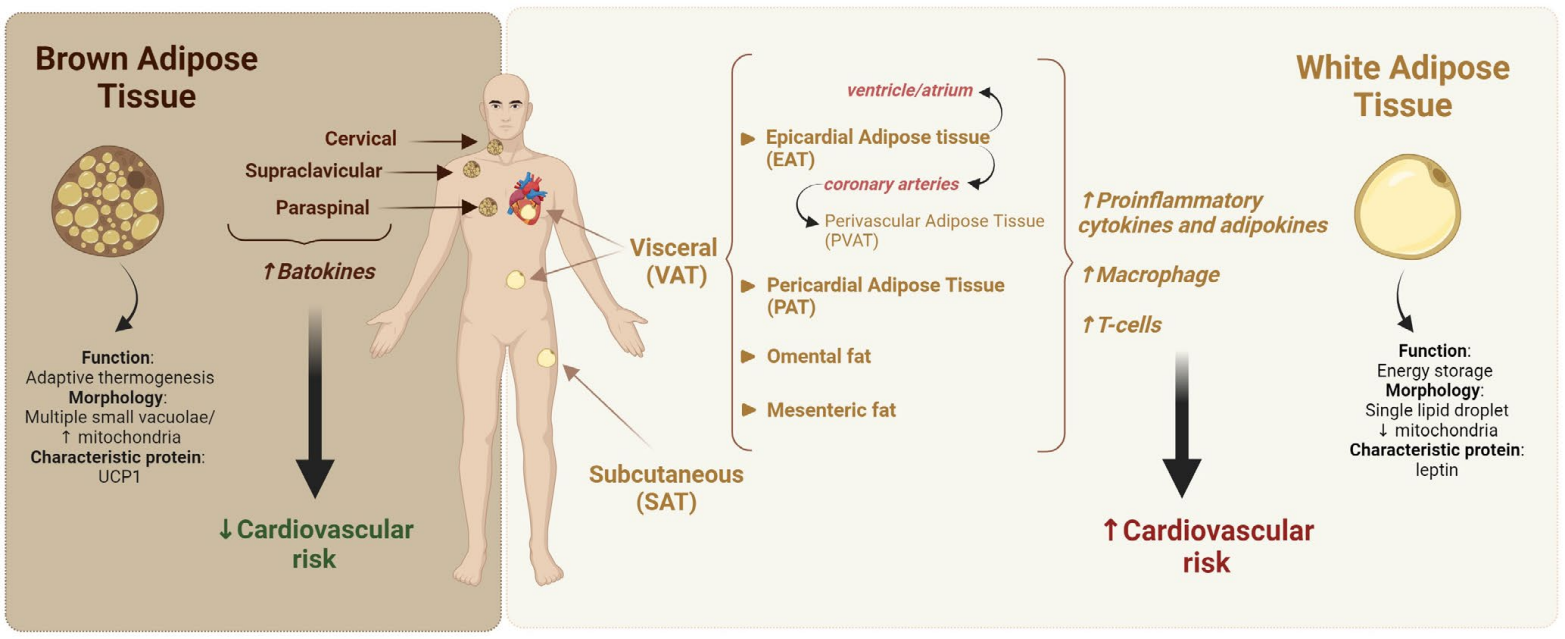
Types of adipose tissue and their main functions and impact on cardiovascular risk. Created by Biorender.com.

The pathophysiology of CV diseases in excess adiposity is strongly influenced by distribution of adipose tissue among main compartments, which are reported to play specific and often opposite roles. White adipose tissue (WAT) is the most abundant adipose tissue in mammals and includes the subcutaneous adipose tissue (SAT), located under the skin, and the visceral adipose tissue (VAT), surrounding abdominal organs (including omental and mesenteric fat) as well as the heart (epicardial and pericardial adipose tissue) and large arteries (Figure 2).^254^, ^255^ Brown adipose tissue (BAT) is less represented (less than 3% of total adipose tissue) and is located in specific body regions, namely paraspinal, supraclavicular and cervical ones.^256^ Conversely, BAT may be associated with protective effects on vessels and heart, that is impaired in obesity.^256^ However, gaps in the definition of obesity and the limited phenotypic characterization of PlwO, as highlighted in the previous section, also reflect inconsistent and even ‘paradoxical’ associations between excess adiposity and CV disease, calling for better designed studies with complete patient assessment.

### 7.2 Summary of the evidence

In PlwO, VAT is associated with the level of oxidative stress, pro‐inflammatory adipocytokines (i.e. leptin and resistin) and cytokines (e.g. IL‐6 and TNF‐α), contributing to vascular dysfunction and hypertension, thereby increasing the risk of symptomatic atherosclerosis.^257^, ^258^, ^259^ The link between BMI and risk of coronary artery disease (CAD) is well established^260^ especially when measures of central adiposity (e.g. WC and WHR) are considered.^261^, ^262^ The time‐dependent nature of obesity's effects on cardiovascular health is evident as BMI‐years and WC‐years are better predictors than BMI and WC alone.^263^ Epicardial (EAT) and pericardial (PAT) adipose tissues are key VAT components affecting cardiovascular health. EAT, particularly perivascular adipose tissue (PVAT), exerts paracrine effects on coronary arteries.^264^, ^265^, ^266^ During obesity, PVAT expansion leads to increased proinflammatory mediators, correlating with atherosclerotic plaque severity. Reducing PVAT volume improves plaque features.^267^, ^268^, ^269^ Additionally, EAT expansion is linked to mitral valve dysfunction and acute CVD.^270^ Obesity also impacts microvascular function, with coronary microvascular disease independently associated with higher BMI. Weight loss improves coronary circulatory function, yet a U‐shaped relationship exists between body weight and myocardial blood flow, potentially reflecting the contrasting effects of visceral and subcutaneous adipose tissue on coronary function.^271^, ^272^, ^273^

VAT contributes to metabolic abnormalities (e.g. elevated cholesterol, triglycerides, glucose and inflammatory cytokines), impairing coronary function and promoting CAD. However, increased subcutaneous adipose tissue in morbid obesity may offer some protection against CAD by reducing lipolysis and modifying adipocytokine profiles.^271^, ^274^ Elevated leptin levels in morbid obesity may aid coronary artery function but also promote left ventricular hypertrophy and fibrosis, worsening cardiovascular outcomes. As coronary circulatory dysfunction has been appreciated as a functional precursor of the initiation and progression of CAD, the reported progressive impairment of coronary circulatory dysfunction from overweight to overt obesity likely reflects a mechanistic connection between dysfunctional coronary arteries and cardiovascular outcome.^275^, ^276^

Beyond ischaemic heart disease, obesity is a major risk factor for heart failure (HF), increasing incidence by 6% per 1 kg/m^2^ BMI increase.^277^, ^278^, ^279^ VAT promotes myocardial fibrosis and hypertrophy, activates the renin‐angiotensin‐aldosterone system, and contributes to insulin resistance and liver steatosis, predisposing to HFpEF over HFrEF. IL‐6 has been implicated in cardiac stiffness and HFpEF severity in PlwO, while HFrEF is typically linked to ischaemic heart disease.^280^, ^281^, ^282^, ^283^, ^284^, ^285^

Obesity also elevates the risk of arrhythmias, accounting for 20% of new atrial fibrillation (AF) cases.^286^, ^287^, ^288^, ^289^, ^290^, ^291^ EAT expansion fosters atrial fibrosis and microvascular dysfunction, linking obesity with AF. A 5 kg/m^2^ BMI increase raises AF risk by 30%, with progression from paroxysmal to permanent AF more likely in obesity.^292^, ^293^, ^294^ EAT correlates more strongly with AF than abdominal obesity, underscoring the role of specific fat compartments in arrhythmogenesis.^295^ Obesity is also a leading non‐ischaemic cause of sudden cardiac death (SCD), linked to ventricular hypertrophy, inflammation and increased neurohormonal activation. PAT strongly associates with SCD risk, infiltrating ventricles, causing fibrosis and facilitating reentrant circuits.^296^, ^297^, ^298^, ^299^, ^300^, ^301^, ^302^, ^303^, ^304^ Additionally, obesity reduces CPR efficacy due to mechanical inertia, ventilation challenges and increased thoracic impedance.^305^, ^306^

### 7.3 Knowledge gap

Despite the considerable progress in the understanding of the pathophysiological mechanisms linking obesity and overweight to CV diseases, a number of questions with unavailable, incomplete, contradictory or biased answers still exist and they affect the day‐to‐day management of obesity, while directly facilitating the spread of weight‐related stigma.^307^ Divergent findings exist regarding the strength of association between obesity and CAD remains, independent of obesity‐related metabolic cardiovascular risk factors. Currently, the most sounded evidence comes from a large meta‐analysis, including 1.8 million individuals and concluding that an optimal treatment of comorbidities, such as hypertension, dyslipidaemia, and glucose disturbances, can reduce by a half the CAD excess risk and by three‐quarters the stroke excess risk associated with high BMI.^112^ Yet, the full benefit was reached only with maintenance of optimal body weight.^112^ Of interest, there is contrasting evidence about the role of metabolic syndrome as a link between obesity and increased CV risk.^147^, ^308^, ^309^ Indeed, many studies showed that obesity without metabolic syndrome does not increase CV risk defining the condition of MHO,^308^ yet this is in contrast with most studies.^147^, ^309^ Similarly, many studies demonstrated such association to be mediated by diabetes, dyslipidaemia or other obesity comorbidities,^263^ while other indicates an independent effect of excess weight on ischaemic heart conditions.^310^, ^311^ As previously detailed, such different findings mostly find explanation in the definition of obesity in the different studies with most of them being based on BMI and not on indices of visceral adiposity. Furthermore, in the plethora of evidence available, even less studies have used direct measures of visceral or epicardial fat by imaging techniques to study the association between obesity and CV disease. Also, assessment of the residual CV risk may not be that accurate when reflecting a single time point rather thana prospective process or when based exclusively on interviews. Overall, this raises an urgent need for large cardiometabolic imaging studies to better understand the specific contributions of various ectopic fat depots to various cardiovascular outcomes. Very much needed as all these adipose depots are correlated with each other, with differences observed among individuals.

Obesity usually associates with a dysfunctional BAT displaying reduced tissue oxidative metabolism (i.e. lower energy expenditure) due to declined hypothalamic stimulation, increased apoptosis and impaired adipocyte recruitment.^312^ Given its atheroprotective role,^313^ such a deteriorated function may have a causative role on the association between obesity and CAD as well as being pursued as a potential therapeutic target.^314^ Yet, how much BAT function contributes to modulate lipid depots and total energy expenditure remains to be elucidated. This is also because the functional study of such adipose compartments requires specific imaging techniques such as PET‐TC scan. Nevertheless, reports suggest that functional BAT (i.e. exhibiting ^18^F‐FDG uptake) associates with reduced VAT mass and hepatic lipid accumulation, along with a lower prevalence of T2D and dyslipidaemia.^315^ Indeed, a healthy BAT can secrete different mediators known as ‘batokines’ which encompass a variety of signalling molecules including peptides, metabolites, lipids or microRNAs exerting different beneficial metabolic effects.^316^ A recent analysis on a large cohort of patients undergoing PET‐CT found an inverse association between the presence of active BAT and diabetes, hypertension, CAD and HF independently from traditional CV risk factors.^256^ Several experimental studies have also suggested the ability of several endogenous and exogenous factors to induce white‐to‐brown adipose tissue conversion. As such, activation of the sympathetic nervous system and its action with adrenergic receptors on white adipocyte membranes has been proposed to trigger a signal transduction cascade that ends in the overexpression of uncoupling protein 1, a protein present in the inner mitochondrial membrane in brown adipocytes and known to play a key role in adaptive thermogenesis and ‘batokines release’.^317^, ^318^ However, the effective WAT browning remains to be fully investigated in human clinical trials as well as the role of ‘batokines’ in promoting cardiometabolic health.^319^, ^320^

With specific reference to heart failure outcome, there might still exist room for an “obesity paradox”. Different studies report better prognosis for HF PlwO rather than those with normal weight. Interestingly, this association is more evident in HFrEF than in HFpEF^321^, ^322^ and remains valid even when indexes of visceral adiposity are taken into consideration.^323^ Of interest, patients with HF are reported to have less amount of EAT and EAT volume seems to inversely correlate with survival.^324^, ^325^ Among the possible explanations is the role of EAT as an energy reserve against cardiac cachexia and sarcopenia in advanced HF.^326^, ^327^ However, how excess adiposity protects long‐term outcome in early stages of HF remains unknown.

Despite recent advancements in obesity therapy showing the efficacy of antidiabetic drugs such as GLP‐1 receptor agonists in reducing MACEs even in euglycaemic PlwO,^328^, ^329^ many open questions in the field of obesity treatment remain. Whether lifestyle modification alone can reduce CVD or mortality among patietns with excess weight has never been proven.^330^ The main limitation of dietary and lifestyle interventions is their poor efficacy in inducing a significant and durable weight loss, as highlighted by the Swedish Obese Subjects trial.^331^ Many studies showed the efficacy of the Mediterranean diet in the general population.^332^, ^333^ Yet not so many reports are available in PlwO, and the results are controversial.^334^, ^335^, ^336^ Similarly, while bariatric surgery is associated with reduced rate of CAD in retrospective and prospective non‐randomized studies,^337^ to date no randomized clinical trials (RCTs) evaluated the effect of such intervention on the incidence of major adverse CV events.^338^

### 7.4 Implementation strategies and potential issues

As mentioned above, the heterogeneous findings in the field of obesity and their relationship with CV diseases suffer from conclusions that cannot be reproduced, or even from studies that arrive at conflicting conclusions. Therefore, the implementation of strategies is urgently needed to standardize definitions, measurements, and terminology to facilitate comparisons among studies. In this sense, data from observational cohorts with short follow‐up periods should be presented as proof‐of‐concept preliminary studies, without supporting overstated conclusions or making inferences about longer follow‐up periods. Similarly, very often studies rely on self‐reported data even for weight and height of patients. While some aspects such as dietary intake and physical activity habits are difficult to assess in a reproducible way, self‐reporting for data that can be measured by researchers should be avoided.

Given the different role played by the different adiposity compartments, much more attention should be paid to the exact definitions of obesity and excess weight. Researchers should refrain from using BMI alone to define obesity while much more attention should be paid to the distribution of fat and metabolic aspects including glycaemic and cholesterol homeostasis. Whenever possible the cohorts should undergo a reproducible characterization of fat depots by imaging techniques. Such an effort will definitively increase the cost of research but will positively impact on knowledge and quality. Another aspect that should be improved involves the employment of causality statement when only associations are reported. In this sense the implementation of RCTs with appropriate control groups will allow us to draw solid conclusions. A global fat assessment, considering the differences shown by different compartments, is often missing in clinical studies. Some heterogeneity in fat characteristics has been already described, suggesting that different degrees of inflammation (both systemic and local) may affect adipose tissue. Potentially, such variability could impact on clinical characteristics of obesities and clinical response to treatment.

The integration of wearable technologies and mobile applications into obesity research represents an opportunity to improve data collection on physical activity, sleep, and dietary habits. Recent advancements in wearable sensors allow for the continuous and objective monitoring of these factors, reducing the reliance on self‐reported data and increasing data accuracy.^339^, ^340^ These technologies can facilitate real‐time tracking of lifestyle behaviours, enabling researchers to obtain more comprehensive insights into obesity‐related health outcomes.^341^, ^342^ Moreover, the development of mobile health applications that integrate data from wearable devices may provide a more detailed assessment of lifestyle factors, supporting more personalized intervention strategies.^343^ However, while wearable technology presents significant benefits, the issue of health data privacy remains a crucial challenge. The collection and storage of sensitive personal data raise concerns about security, data sharing and patient consent. Ensuring compliance with regulatory frameworks and implementing robust data protection measures is essential to maintain user trust and ethical research practices.^344^ Future research should aim to address these challenges while leveraging wearable technology to enhance the accuracy and reproducibility of obesity‐related studies.

## 8 TASK 3. POINT BY POINT SUMMARY

**Table:** 

Knowledge gaps	Gaps in obesity knowledge make obesity‐related communication less clear, directly facilitating the spread of weight‐related stigma The degree to which obesity's correlation with CAD remains unaffected by metabolic cardiovascular risk factors associated with excessive weight remains unclear The prognostic value of MHO remains unknown The role of brown adipose tissue and its potential as therapeutic target to reduce obesity‐related CV burden remains mostly unexplored Does the obesity paradox for HF outcome really exist? How does excess adiposity protect long‐term outcome in early stages of HF? Components of lifestyle modification should be better studied: physical activity, exercise, overall food‐based diet quality, sleep
Implementation strategies	Uniform definitions, measurements, and terminology to facilitate comparison among studies are needed Results from observational cohorts with short follow‐up periods should not support overstated conclusions or infer about longer follow‐up periods The use of self‐reported data should be limited to measurements that cannot be obtained otherwise Refrain from using BMI alone to define obesity. Emphasize the distribution of fat (visceral obesity) and metabolic aspects including glycaemic and cholesterol homeostasis The use of imaging techniques to quantify/characterize fat depots should be encouraged Causality statements should be avoided when reporting association data Consider the benefits of non‐dieting approaches to treat obesity
Potential issues	Higher cost for imaging techniques to provide better fat depots characterization Exclude the confounding factors to properly identify, in carefully designed studies, the existence of paradoxical obesity

Abbreviations: BMI, body mass index; CAD, coronary artery disease; CV, cardiovascular; HF, heart failure; MHO, metabolically healthy obesity.

## 9 TASK 4. METABOLIC DYSFUNCTION‐ASSOCIATED STEATOTIC LIVER DISEASE (MASLD): MANAGING THE WHOLE BY PREDICTING THE PARTS

### 9.1 Background

The huge social, economic and health burden associated with obesity is also due to an increased prevalence of steatotic liver disease that globally increased from 25.2% in 1990–2006 to 38% in 2016–2019.^345^ The rise is evident in all age classes, including children and adolescents, making steatotic liver disease the most frequent chronic liver condition today.^346^, ^347^, ^348^, ^349^ In addition, liver steatosis is no longer considered a mere ‘fellow traveller’ with metabolic disorders, as it independently increases the risk of atherosclerosis,^350^ CV disease^351^ and cancer, the latter mainly affecting the digestive system and the lungs.^352^, ^353^ The term ‘fatty liver hepatitis’ first appeared in 1962 in the German literature while the term ‘non‐alcoholic steatohepatitis’ was coined in 1980, defined by the histopathological hallmarks of steatosis, lobular inflammation and liver cell damage.^354^ Since 1980, the term non‐alcoholic fatty liver disease with its acronym NAFLD has been adopted to define the presence of steatosis in at least 5% of hepatocytes by histology or at least 5.5% by magnetic resonance spectroscopy in individuals with no or little alcohol consumption.^355^ Accepted cut‐off of daily alcohol intake was ≤20 g in females and ≤30 g in males. Other causes of chronic liver disease had to be absent, including alcohol use disorder, viral hepatitis, genetic defects and drug‐induced liver injury.^356^, ^357^ The spectrum of NAFLD included subgroups of patients along a sequence of potential progression from non‐alcoholic fatty liver or NAFL (histologically characterized by macrovescicular steatosis and mild lobular inflammation)^358^ to non‐alcoholic steatohepatitis or NASH (additionally featuring hepatocellular ballooning with more severe inflammation and a variable degree of fibrosis) to liver cirrhosis and hepatocellular carcinoma (HCC). In the past few years, a change in NAFLD terminology has been the focus of active discussion with a goal to move from a diagnosis of exclusion to a pro‐active definition rooted in disease pathophysiology. The term ‘metabolic dysfunction‐associated fatty liver disease’ (MAFLD)^359^, ^360^ was introduced in 2020 followed by the term ‘metabolic dysfunction‐associated steatotic liver disease’ (MASLD)^361^ proposed by a large, global consensus panel in 2023. Besides pathophysiological considerations, a major intention behind these changes was to remove the potentially stigmatizing terms ‘alcoholic’ and ‘fatty’ from the disease terminology.^362^ Broad acceptance of the new nomenclature will take some time as it has an impact on public perception, culture, research and previous scientific literature accumulated for NAFLD over the years.^363^, ^364^, ^365^ Furthermore, albeit developed to provide a more accurate reflection of the disease's metabolic basis, there are challenges in the application on MASLD definition, particularly concerning overlaps and distinctions between these classifications. This underscores the need for careful consideration when implementing new diagnostic criteria to ensure clarity and consistency in clinical practice.^366^

### 9.2 Summary of evidence

**FIGURE 3:**
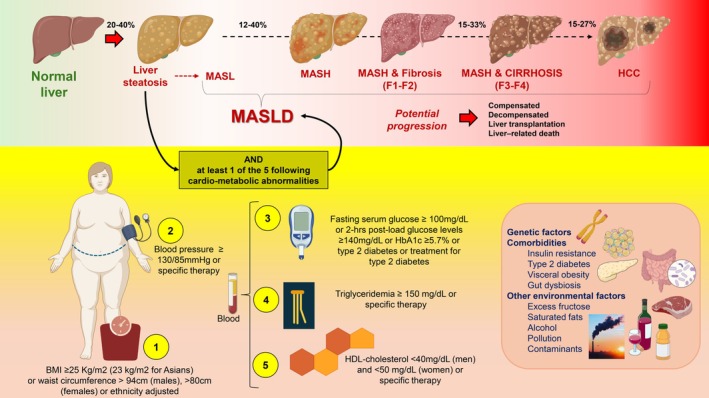
The novel criteria for the diagnosis of metabolic dysfunction‐associated steatotic liver disease (MASLD). The presence of liver steatosis by imaging and the presence of at least one cardio‐metabolic abnormality among five is required. Once the diagnosis of MASLD has been established, the potential progression of disease includes metabolic dysfunction‐associated steatohepatitis (MASH) without and with fibrosis, cirrhosis and hepatocellular carcinoma (HCC). The continuous role of genetic factors, comorbidities, and other environmental factors is also depicted in the appropriate box. This cartoon depicts several close interactions between the condition of liver steatosis and the development of chronic non‐communicable diseases and vice versa. Created by Biorender.com.

It is expected that the new MASLD nomenclature will facilitate efforts to refine the pathophysiological framework of disease heterogeneity, introduce preventive strategies and develop clinical care pathways for affected individuals, including the assessment of long‐term health risks associated with various trajectories of the disease. This phenotype‐oriented assessment should not only focus on liver disease severity (i.e. staging and grading steatohepatitis, fibrosis, cirrhosis and liver cancer), but should also consider systemic conditions related to metabolic dysfunction (e.g. risk of cardiovascular disease, T2D and cancer). The novel definition of MASLD includes any condition of liver steatosis diagnosed by serum biomarkers, imaging or liver histology, if associated with at least one out of five cardiometabolic risk factors (Figure 3).^361^ MASLD represents a sub‐category within the larger umbrella term ‘steatotic liver disease’, a general condition encompassing all liver diseases characterized by abnormal liver fat accumulation.^363^ What is still missing in the new nomenclature, however, is a corresponding term to NAFL (a histologically classified state without steatohepatitis hallmarks). Although NAFL of ‘simple steatosis’ may generate limited interest in the identification and general management of disease, there is increasing evidence that preclinical stages of liver fat accumulation is a key determinant of subsequent development of fibrosis, cirrhosis and HCC,^367^, ^368^ indicating a need for early identification of patient sub‐groups at increased risk of unfavourable disease outcomes.^369^ Further adjustments in MASLD terminology are expected to allow for a more granular and precise categorization of patients with steatotic liver and metabolic disorders, paving the way to personalized therapeutic approaches. The new nomenclature, however, may facilitate research of pathogenic pathways linking fat over‐storage in hepatocytes to a variety of systemic, noncommunicable diseases. This approach will ultimately bring relevant elements into a comprehensive estimate of health risk in subjects with MASLD. It is encouraging that there is already evidence from comparative studies for a near‐perfect overlap of populations categorized as MASLD or NAFLD, which may allow the incorporation of the immense knowledge accumulated on NAFLD in the past 40 years.^365^, ^370^, ^371^, ^372^, ^373^ MASLD represents a common liver condition where both external and inherited risk factors trigger the disease and modulate its progression.^374^ Although external factors predominantly influence MASLD, genetic analyses of patients with steatotic liver disease have shown that inherited predisposition also plays a significant role in the development and progression of hepatic steatosis.^369^ For example, twin studies demonstrated not only a strong concordance of steatosis and fibrosis in monozygotic twins^375^ but also underscored a major shared genetic effect on these two phenotypes.^376^ In the past two decades, numerous studies on adult and paediatric patients with fatty liver have demonstrated that carriers of the common PNPLA3 (patatin‐like phospholipase domain‐containing protein, also known as adiponutrin) variant p.I148M are at increased risk of developing hepatic steatosis,^377^, ^378^, ^379^ fibrosis and cirrhosis,^380^ as well as HCC.^381^ These associations extend to liver diseases beyond MASLD,^382^, ^383^ rendering PNPLA3 p.I148M a common genetic modulator of severe liver phenotypes in patients with chronic liver conditions.

In addition to PNPLA3 p.I148M, other variants such as MBOAT7 (membrane‐bound O‐acyltransferase domain‐containing protein 7) p.G17E, TM6SF2 (Transmembrane 6 superfamily 2) p.E167K and APOE rs429358 (apolipoprotein E) have also been shown to increase the risk of liver injury in patients with MASLD and alcohol‐associated liver diseases.^384^, ^385^, ^386^ Given the central role of PNPLA3 in the progression and development of MASLD, numerous studies have been conducted to elucidate the function of this protein, particularly the effects caused by the p.I148M minor allele. In brief, adiponutrin is expressed on intrahepatic lipid droplets, where it might exert lysophosphatidic acyltransferase^387^ or lipase^388^ activities. Recent analyses highlighted its role in mitochondrial function and implicated its involvement in de novo lipogenesis.^389^ Regarding other polymorphisms involved in modulating the MASLD phenotype, TM6SF2 plays a role in VLDL (very low‐density lipoproteins) secretion.^390^ Carriers of the TM6SF2 p.E167K variant have an increased risk of hepatic steatosis, but this variant also appears to have certain cardioprotective effects.^391^ The MASLD‐modulating MBOAT7 SNP is associated with lower expression of the protein,^392^ which seems to increase liver injury.^393^ In addition to harmful variants that enhance liver injury in patients with MASLD, some protective polymorphisms have been detected. For example, SNPs in the MTARC1 (mitochondrial amidoxime‐reducing component 1) and HSD17B13 (17β‐Hydroxysteroid dehydrogenase type 13) genes reduce the risk of developing severe MASLD phenotypes.^394^, ^395^, ^396^, ^397^ Given these complex associations between several genetic variants and MASLD, the use of polygenic risk scores (PRS),^398^ along with the inclusion of external modifiers, can help precisely delineate the trajectory of progressive fatty liver in each patient.^369^ Specific fatty liver alleles are surprisingly frequent in the general population with a wide range of minor allele frequency of the PNPLA3 p.I148M risk allele (rs738409) ranging from 8% in Kenya up to 72% in Peru.^399^ As much as 50% of adults with MASLD carry at least one copy of this risk allele (rs738409).^400^ The thrifty gene hypothesis represents an approach to explain why unfavourable genes involved in metabolism that evolved for survival in the late Palaeolithic era are so frequent today. Advances in ancient DNA technology now enable studying natural selection by analysing samples spanning the last 50,000 years. Recent data indicate that archaic humans like Neanderthal and Denisovan exclusively carried the PNPLA3 risk allele, suggesting fixation of the PNPLA3 risk allele in the ancestor of all archaic humans.^401^ Analysis of its further trajectory over the last 15,000 years of modern human history revealed that ancient allele distributions roughly match the distribution we observe today. Data from logistic analyses either through time or along latitude do not support a significant contribution of natural selection, even though a generally observed decrease in frequency over time may still be suggestive of moderate selection.^401^ Given the limited lifespan of archaic and early modern humans, it is at least not surprising that no signals towards negative selection exists. From these archaeogenetic data, we can conclude that the ability to store fat was likely an advantage throughout most of human history, particularly for Neanderthals under ice age conditions. Supportive of this hypothesis is recent data from the Yakut population in the coldest northeast region of Russia, where the PNPLA3 variant allele is predominant in almost 90% of the population.^402^ Further research is needed to fully understand the impact of these exciting observations on MASLD heterogeneity and on developing targeted management strategies.

Based on individually inherited predisposition, onset and progression of MASLD largely depend on external factors leading to metabolic dysfunction both at systemic and hepatic levels.^362^ Excessive hepatocellular accumulation of lipids, primarily driven by excessive caloric intake in MASLD and mainly occurring in the form of triglycerides, is a consequence of imbalance between lipid input and output, exemplified by increased de novo lipogenesis versus increased efflux of free fatty acids (FFAs), which are the main substrate for triglyceride synthesis by esterification.^403^ Over‐storage of FFAs in the liver generates a cascade of negative pathways which includes lipotoxicity,^404^, ^405^, ^406^ mitochondrial and endoplasmic reticulum dysfunction,^407^ activation of signalling pathways related to metabolism and inflammation,^408^ and receptor activation which will promote inflammation.^409^ Genetic polymorphism and pathologic gene variants amplify or mitigate the environmental pressure leading to caloric imbalance and may point to specific pathways and molecular targets that may be utilized for pharmacological interventions in clusters of patients with different MASLD phenotypes.^362^ Besides deranged lipid metabolism, biochemical alterations in MASLD also include deranged carbohydrate metabolism, with increased levels of enzymes governing glycolysis as hexokinase 2 and pyruvate kinase isozyme type M2^410^, ^411^ and with hepatic insulin resistance.^412^ The suppression of hepatic insulin clearance critically drives hyperinsulinaemia. This suppression, resulting from impaired activity of the insulin‐degrading enzyme, leads to increased hepatic CD36 expression, which enhances the uptake of circulating free fatty acids and further aggravates hepatic steatosis.^413^, ^414^, ^415^

Furthermore, deranged bile acid homeostasis^416^ has critical effects on the function of the nuclear receptor farnesoid X receptor (FXR),^417^, ^418^ and on the membrane‐associated G protein‐coupled bile acid receptor 1 (GPBAR‐1),^419^, ^420^ during bile acid absorption in the terminal ileum.^417^ In the liver, FXR deactivates hepatic lipogenesis by inhibiting SREBP1c, promotes FFA β‐oxidation by activating peroxisome proliferator‐activated receptor‐α (PPARα), and facilitates the clearance of VLDL in plasma by regulating microsomal triglyceride transfer protein (MTTP), ultimately improving metabolic dysfunction in MASLD.^421^, ^422^, ^423^ Furthermore, the activation of intestinal FXR releases the enterohormone fibroblast growth factor 19 (FGF19) from human enterocytes. FGF19 entering the liver via the portal tract, can protect from hepatic steatosis.^424^, ^425^ Of note, both GPBAR1 and FXR are expressed in the aortic wall, with regulatory functions on the interaction between endothelial cells and macrophages, and a critical role in the maintenance of cardiovascular homeostasis.^426^ In apolipoprotein E^−/−^ and wild‐type mice, the dual GPBAR1/FXR agonist BAR502 decreased plasma levels of cholesterol and low‐density lipoprotein, mitigated the development of both liver steatosis and aortic plaque formation, and shifted the polarization of circulating leukocytes towards an anti‐inflammatory phenotype.^426^ Other nuclear receptors involved in the maintenance of metabolic homeostasis and in the pathogenesis of liver steatosis are the liver X receptor (LXR),^427^ the pregnane X receptor (PXR)^428^ and the vitamin D receptor (VDR).^429^ Dysfunctional nuclear receptors contribute to the development of liver inflammation and to the activation of local and systemic inflammatory pathways. In the liver, an overexpression of the LXRα gene and its lipogenic targets PPAR‐γ (peroxisome‐proliferator‐activated receptor‐γ), SREBP (sterol regulatory element‐binding protein)‐1c, SREBP‐2 and FAS (fatty acid synthase) has been reported in subjects with steatosis.^430^ More recently, LXR has been indicated as a key regulator of lipid metabolism in human aortic endothelial cells, and as a potential target in cardiovascular diseases.^431^ Similarly, PXR is also expressed in human vessels (endothelial and smooth muscle cells)^432^ and platelets.^433^ PXR ligands generate anti‐atherosclerotic effects increasing cholesterol clearance and HDL production in animal models^434^, ^435^ and, in humans, decrease platelet functions with anti‐thrombotic effects.^433^ VDR is also expressed by endothelial cells, vascular smooth muscle cells and cardiomyocytes, playing a role in the development of atherosclerosis and cardiovascular diseases.^241^ Finally, thyroid hormone receptors (THR) form homodimers with other nuclear receptors and the particularly liver‐specific THRβ modulates the expression of relevant genes in cholesterol and fatty acid metabolism.^436^

Alterations of the gut microbiota generated by over nutrition^437^ can also play a major role and gut dysbiosis can predispose to the onset and development of MASLD,^438^, ^439^, ^440^, ^441^, ^442^ mainly through the production of steatogenic inflammatory mediators, including endotoxin^443^ or reduced production of protective short‐chain fatty acids (SCFAs).^444^ Microbial products can play a critical role, in the context of high‐fat diet, when endotoxin level is increased.^445^, ^446^ SCFAs, by contrast, generate beneficial metabolic effects that preserve intestinal permeability and enhance insulin secretion and sensitivity by increasing the secretion of glucagon‐like peptide‐1 (GLP‐1) and peptide YY (PYY).^447^, ^448^, ^449^ SCFAs mitigate chronic inflammation by activating immune cells, specifically regulatory T cells (Tregs) on the one hand^450^ but specifically faecal propionate and acetate levels were also associated with lower rTregs and higher Th17/rTreg ratio in MASH patients.^451^ Decreased production of SCFAs secondary to gut dysbiosis increases the severity of MASLD^452^ in combination with a decreased relative abundance of Faecalibacterium prausnitzii, Akkermansia Muciniphila and Dysosmobacter welbionis. Gut dysbiosis can also impair the release of a lipoprotein lipase inhibitor, fasting‐induced adipose factor (FIAF), from the intestinal epithelium, with subsequently increased levels of FFAs in the liver.^438^ Gut dysbiosis, particularly when characterized by increased relative abundance of Enterobacteriaceae, Escherichia coli and Shigella, has been linked with the progression of MASLD.^453^, ^454^ In apolipoprotein E^−/−^ and wild‐type mice, the combined administration of BAR502 (a dual GPBAR1/FXR agonist) counterbalanced the intestinal dysbiosis and modulated the synthesis of bile acids, with a shift in the pool composition towards FXR antagonists and GPBAR1 agonists, and beneficial metabolic effects.^426^ Besides pathogenetic mechanisms leading to different obesity phenotypes^455^ and to the onset and development of MASLD,^362^, ^442^ the presence of gut dysbiosis also promotes the progression of atherosclerosis,^350^, ^456^ mainly through increased production of trimethylamine N‐oxide (TMAO) and alterations of the gut barrier, with lipopolysaccharide (LPS) translocation into the bloodstream.^350^ A recent study in humans with liver steatosis associated with metabolic diseases identified characteristic changes in stool microbiomes and plasma microbial metabolites linked with indices of cardiovascular risk as the intima‐media thickness and lipid profile.^457^ The pathophysiology of the disease provides multiple potential targets for drug treatment which are currently tested in Phase II and III.^458^, ^459^ As such, the thyroid hormone receptor beta agonist resmetirom has recently been licensed by the FDA as the first drug for the treatment of MASH.^460^

Based on heritable gene polymorphisms, the pathogenesis of MASLD is also affected by epigenetic mechanisms (i.e. histone acetylation, small and not‐coding RNA, DNA methylation, chromatin remodelling).^461^, ^462^ These mechanisms regulate gene expression in response to a number of external variables as diet,^463^, ^464^, ^465^ physical activity^465^, ^466^, ^467^ and environmental exposure to toxic chemicals (e.g. endocrine disrupting chemicals,^468^, ^469^ perfluorooctanesulfonate,^470^ heavy metals^471^). This epigenetic modulation affects the expression of specific individual phenotypes in subjects with liver steatosis and altered metabolic homeostasis,^461^ also with transgenerational effects.^464^, ^472^, ^473^, ^474^ DNA methylation is deeply involved in the pathways linking accumulation of saturated fatty acids in the liver and metabolic dysregulation in subjects with steatosis.^475^ A relevant role is also played by the composition of gut microbiota^476^, ^477^ that can generate host epigenetic variations affecting the development of MASLD and a possible progression towards hepatocellular carcinoma.^476^ DNA methylation affecting the hepatic expression of dipeptidyl peptidase 4 (DPP4) can worsen the development of liver steatosis affecting hepatic insulin signalling and decreasing GLP‐1 levels.^478^, ^479^ The hypermethylation of the NF‐E2‐related factor 2 (Nrf2) gene promoter can be mediated by high‐fat diet and modulates the expression of lipogenic genes.^480^ Similarly, DNA methylation levels of the fatty acid desaturase 2 (FADS2) gene in the liver, that encodes delta‐6 desaturase, significantly affects the development of liver steatosis and its progression towards steato‐hepatitis.^481^, ^482^

The natural history of MASLD indicates that different subgroups of patients are predisposed to different risks for major liver‐related outcomes. While the presence of MASLD has been linked to increased risk for all‐cause mortality,^483^, ^484^ cardiovascular disorders^351^, ^485^, ^486^ and for liver and extra‐hepatic cancer,^352^, ^353^ patient subgroups in which a specific assemblage of risk factors may determine the magnitude of this association are not fully characterized.^487^, ^488^, ^489^ Increasing severity of MASLD may be one such determinant. In a large cohort of patients observed during a median follow‐up of 27 years, the coexistence of MASLD and advanced fibrosis was associated with an increased risk of all‐cause mortality, as compared to MASLD without advanced fibrosis.^488^ All‐cause mortality in MASLD seems unchanged after adjustment for demographic factors, body mass index and the presence of chronic hepatitis B or C. In addition, a significant association emerged between MASLD and concomitant alcohol consumption in MASLD (i.e. MetALD) resulting in increased all‐cause mortality.^490^, ^491^

Recent research using cluster analysis methods to identify subgroups of patients with obesity^492^ and diabetes^349^, ^493^ provided a blueprint for similar studies to define patients clusters in which MASLD phenotypes are associated with markedly different risks of major liver outcomes and association with adiposopathy, diabetes, and cardiovascular disease.^494^, ^495^, ^496^, ^497^, ^498^ While these studies represent a snapshot of cross‐sectional data within the natural history of MASLD, they are nevertheless an essential step towards identifying distinct disease trajectories with specific drivers of liver and non‐liver outcomes within each cluster, identifiable by a combination of genetic variants with clinically significant features and comorbidities. While it may seem impractical to consider genetic studies to screen for potentially pathogenic variants in a large population, one would^499^ hope that clinical and laboratory parameters, lifestyle elements, health risk behaviours and other socio‐economic factors will give early clues for increased risk of MASLD progression and signal the need for more detailed investigation and specific intervention at the individual level.^500^, ^501^, ^502^ This task may be further refined by using the recently described method of concordant phenotypic profiling to analyse intra‐cluster heterogeneity and identify individual outliers in need of enhanced attention.^503^, ^504^ Time‐series clustering such as group‐based trajectory models and hidden Markov models may prove useful in mapping progression and accounting for transition between clusters.^496^, ^505^

By definition, subjects with MASLD suffer from metabolic dysfunction that may also manifest as obesity, T2D, MetS, chronic kidney diseases, altered serum lipid profile or arterial hypertension^506^ and carry a higher risk of fatal and/or non‐fatal CV events after adjusting for other CV risk factors.^485^, ^486^, ^507^ Indeed, the presence of liver steatosis has been linked to increased risk for CV mortality^508^ and the American Heart Association identified liver steatosis as an independent factor for CV diseases.^351^ Based on the substantial heterogeneity of MASLD, it is also reasonable to conceive subgroups of patients with different risk for major non‐liver outcomes including CV disease. A Korean study divided 9497 participants in MASLD, MASLD with an increased alcohol intake (MetALD) and alcohol‐associated liver disease (ALD). In this cohort, the risk of CVD increased in the order of no SLD, MASLD and MetALD.^509^ We may therefore need to consider the screening of subjects with MASLD for CV diseases and, conversely, the screening of subjects at increased CV risk for MASLD. Indeed, the arena of specialists involved with the idea of ‘liver steatosis in mind’ is expanding and will impact large groups of populations worldwide, although one needs to recognize the potentially different pace of disease progression and time frame of complications in CV versus liver disease. The risk for CV mortality and major adverse cardiovascular events is also elevated in lean subjects with MASLD, who present a 50% increase in CV mortality odds, as compared with non‐lean MASLD.^510^ Higher liver fat content (assessed with magnetic resonance imaging proton density fat fraction) increases CV risk independently of age, gender, ethnicity and BMI.^511^ Furthermore, available evidence points to a major role in modulating CV risk played by hepatic fibrosis.^64^, ^512^, ^513^, ^514^ In fact, liver fibrosis has been linked with CV risk,^515^ atherosclerosis,^512^, ^514^ structural left ventricular remodelling,^516^ HF^517^ and CAD.^513^

Future studies should focus the CV risk on different phenotypes of patients with MASLD. In this respect, a recent model based on microbial metabolites and nine clinical parameters was able to accurately predict patients at cardiovascular risk in a population of subjects with liver steatosis associated with metabolic diseases. The detection of risk occurred at a very early stage, before the formation of pathological changes of the carotid artery.^457^

MASLD has long been associated with an increased risk of primary liver cancer.^518^, ^519^, ^520^ Association of MASLD with T2D is a major risk factor for the development of HCC.^521^ Additional evidence also points to a credible association of MASLD with extrahepatic cancers.^352^, ^353^, ^522^, ^523^, ^524^, ^525^, ^526^ A recent systematic review and meta‐analysis confirmed the association between MASLD and extrahepatic cancer in the stomach, colon, rectum, pancreas, biliary duct, thyroid, urinary system, breast, skin and female genitals.^527^ A large cohort study confirmed the link between liver steatosis and cancer risk (mainly digestive system and lung cancers), and showed significant association with the duration of steatosis (i.e. the younger the onset age of steatosis, the greater the cancer risk).^352^ This last aspect is particularly relevant due to the increasing epidemiologic burden of MASLD in children and adolescents,^346^ and to the progressive increase in lifespan.^528^

There is little known about the potential association between different subgroups of patients with MASLD and the risk of HCC and various types of non‐liver cancers. In the case of HCC, this matter is further complicated by its emergence in noncirrhotic MASLD, which occurs at a low rate yet becomes non‐negligible due to high MASLD prevalence.^529^ Polygenic risk scores and clinical scores have both been developed to predict noncirrhotic HCC^398^, ^488^ but show low sensitivity and further work is needed to resolve this important public health challenge. A Korean nationwide cohort study^530^ showed that the risk of extra‐hepatic cancer was higher when fatty liver disease (defined as MAFLD) was diagnosed in lean subjects or it was associated with T2D when compared with T2D without liver steatosis.^530^ However, this was not the case in overweight/obese patients, whose risk of extrahepatic malignancy was similar regardless of the presence or absence of fatty liver disease.^530^ Another large study showed that the coexistence of MASLD and T2D significantly increases the risk of HCC as well as pancreatic, breast and renal cancer.^525^ Further studies will be needed to determine the extent to which MASLD may increase the risk of various types of cancer in its own right and in association with other diseases of metabolic dysfunction such as T2D.

### 9.3 Implementation strategies and potential issues

The extraordinarily high prevalence of MASLD in the general population calls for efforts to understand the causal factors and pathogenic mechanisms of this complex disorder with highly diverse outcomes. Depending on the constellation of risk factors and associated individual phenotypes in MASLD, management strategies—beyond universally promoting comprehensive lifestyle interventions—may widely differ. MASLD has been associated with increased risk of both liver‐related and all‐cause mortality, while there is an unmet need to identify subgroups of patients at risk and in need of targeted interventions to halt or delay progression of liver disease and associated metabolic comorbidities. Limited feasibility and substantial healthcare costs dictate reliable and timely risk assessment for the accurate selection of patients who could benefit the most from enhanced monitoring and advanced therapeutic options such as liver‐directed and weight management pharmacotherapy. In particular, a comprehensive classification of subgroups and patients with MASLD should account for the presence of various cardiometabolic risk factors and comorbidities, coexisting liver disease (mainly viral hepatitis^531^, ^532^ and autoimmune hepatitis^533^), genetic predisposition, unhealthy dietary and other lifestyle habits,^1^, ^534^ socio‐economic factors^535^ and exposure to a complex mixture of environmental toxins in an obesogenic world.^1^, ^536^, ^537^

Unhealthy dietary habits play a central role in the development and progression of MASLD. Specifically, high intake of ultra‐processed foods and sugary drinks has been strongly associated with an increased risk of hepatic steatosis, insulin resistance and systemic inflammation, which contribute to liver disease progression. Ultra‐processed foods, characterized by high levels of refined sugars, unhealthy fats and artificial additives, promote metabolic dysfunction by altering gut microbiota composition and increasing hepatic fat accumulation.^538^ Likewise, sugary drinks, which are major sources of added fructose, drive hepatic de novo lipogenesis, exacerbating lipid accumulation in the liver and fostering oxidative stress.^539^ The pervasiveness of these dietary components in modern diets underscores the need for targeted interventions aimed at reducing their consumption. Public health initiatives promoting awareness and behavioural changes, such as taxation on sugar‐sweetened beverages, clearer food labelling and education campaigns, could help mitigate the dietary risk factors contributing to MASLD.^540^

Furthermore, although the term MetALD points to individuals with MASLD who are concomitantly affected by moderate alcohol consumption, there is a great need for a more detailed characterization of these subjects,^442^ both in terms of liver disease progression and of the individual risk for extra‐hepatic events. In fact, a dynamic change in the classification of a patient as having MASLD, MetALD or ALD is possible throughout life depending on the evolution of metabolic dysfunction and variable alcohol consumption over time against an individual background of genetic polymorphisms regulating the metabolism of alcohol. Although additional classification of subjects with MASLD to identify disease clusters is strongly needed, it is important to mitigate the risk of misclassifications, unnecessary confusion or inappropriate definitions while incorporating new information within the framework of steatotic liver disease. Further studies will determine how emerging criteria for the subclassification of MASLD will contribute to increased awareness of the disorder, and how a more accurate definition of at‐risk patient subgroups will facilitate better understanding of the pathophysiology, allow for the design of successful pharmaceutical trials and promote effective strategies for the prevention and management of divergent disease trajectories. Finally, it will be important to determine how early detection of increased risk of disease progression alters liver and non‐liver associated mortality in MASLD. Large population‐based studies indicate that the vast majority of patients with MASLD‐associated cirrhosis are first diagnosed with a decompensation event.^541^ Moreover, MASLD has been associated with a more than 4‐fold increased likelihood of having unrecognized cirrhosis compared to chronic hepatitis C.^542^ These findings suggest that early diagnosis and risk stratification may be necessary in MASLD once we have evidence that timely detection of liver disease alters the morbidity and mortality associated with liver disease and once we know the efficacy of our interventions by the stage of MASLD detected.^543^

## 10 TASK 4. POINT TO POINT SUMMARY

**Table:** 

Knowledge gaps	Comprehensive analysis of different pathogenic pathways involved in MASLD in individuals with normal weight compared to those living with overweight or obesity Characterization of personalized therapeutic approaches based on distinct MASLD phenotypes Integration of studies using metagenomics, transcriptomics, proteomics and metabolomics to assess the role of individual confounders in various outcomes of MASLD
Implementation strategies	Identification of specific links between causal factors, pathogenic mechanisms and diverse outcomes of MASLD Precise identification of different health risks and outcomes according to the various phenotypes of MASLD and associated comorbidities Accurate characterization of alcohol consumption and impact on disease outcomes in subjects with MetALD Assessment of the role of gut microbiota in shaping the individual metabolic phenotype that predisposes individuals to MASLD Identify the specific root connecting obesity to the onset and development of MASLD
Potential issues	There is a need for a multidisciplinary, integrated approach that combines distinct skills to achieve multi‐omics phenotyping and a comprehensive long‐term risk analysis of MASLD Consider the variable gene–environment interactions, epigenetic mechanisms, and the role of environmental factors (including pollution) in the onset and progression of MASLD Create knowledge and awareness about the role of dietary pattern in the development of MASLD

Abbreviations: MASLD, metabolic dysfunction‐associated steatotic liver disease; MetALD, metabolic dysfunction and alcohol‐related liver disease.

## 11 TASK 5. METABOLIC COMPLICATIONS OF PAEDIATRIC OBESITY: CURRENT KNOWLEDGE AND RESEARCH GAPS

### 11.1 Background

Early‐onset obesity is an increasingly concerning public health problem that predisposes children and adolescents to numerous cardiometabolic complications throughout their life. Although some progress in the understanding of early onset obesity has been made, its pathogenesis remains largely elusive. Obesity in paediatric age is associated with metabolic complications with a common culprit: insulin resistance. In fact, in PlwO, due to the inability of adipose tissue to expand indefinitely, an excess of FFA is released from the adipocytes and accumulates within other tissues, such as muscle and liver. This massive exposure of muscle and liver to FFA results in an overproduction of diacylglycerol (DAG) and ceramides, that affect insulin signalling and reduces the suppression of gluconeogenesis and glycogenolysis. When the adipose tissue becomes resistant to insulin adipose tissue lipolysis is no longer properly suppressed. This results in an additional increase of FFA flux from adipose that further worsens the ability of insulin to regulate glucose and lipid metabolism. The consequence of these metabolic changes is the high prevalence among youth living with obesity of conditions driven by IR, such as prediabetes (~20%), fatty liver disease (~30%), T2D (~5%) and MetS (~40%). Furthermore, IR has been shown to worsen endothelial function since childhood, leading to an increased risk of developing cardiovascular diseases.^544^ Several therapeutic and preventive strategies have been proposed to treat obesity or at least to halt its progression, although high dropout and failure rates have been observed. The recent GLP‐1 analogues (namely liraglutide and semaglutide) approved in paediatrics to treat obesity are characterized by about 25% failure rate. Furthermore, side effects are often observed during these treatments and the need for long‐term use can represent a significant economic burden for the families of patients.

### 11.2 Summary of Evidence

Obesity is associated with profound modifications in the structure and function of adipose tissue, leading to significant changes in both the intracellular and extracellular environments, including alterations in the extracellular matrix. In children living with obesity, excess caloric intake leads to an increase in both the number of adipocytes (hyperplasia) and their size (hypertrophy). Hyperplasia is driven by progenitor cells, known as preadipocytes, that differentiate into mature adipocytes to store triglycerides. On the other hand, hypertrophy results from abnormal triglyceride accumulation, often leading to dysfunctional adipocytes, which undergo early cell death and trigger sterile inflammation within the adipose tissue.^545^ This inflammatory process is observed in both youth and adults living with obesity and is characterized by an increased production of pro‐inflammatory cytokines and mediators. Moreover, the macrophage population within the adipose tissue increases from 10% to 41%.^546^ Histological evidence demonstrates that these macrophages accumulate around dying adipocytes, forming, in some cases, crown‐like structures that are associated with a worsened metabolic phenotype.^547^ Although adipose tissue inflammation in obesity has been well‐documented for over a decade, the specific nature, morphology and function of the macrophages involved remain unclear. While most macrophages in inflamed adipose tissue are thought to be of the M1 type, recent research has suggested the existence of a unique population of metabolically activated macrophages that contribute to inflammation by secreting pro‐inflammatory adipokines and reducing the production of anti‐inflammatory adipokines.^546^ As obesity progresses, adipose tissue inflammation leads to increased synthesis of extracellular matrix components, particularly Collagen VI, which appears to play a key role in adipose tissue fibrosis.^548^, ^549^, ^550^ Adipose tissue fibrosis has emerged as a significant factor contributing to the progression of obesity‐related complications by impairing the ability of adipose tissue to expand, thereby exacerbating IR.^549^ Recent studies in both adults and adolescents have highlighted that fibrosis, rather than the capacity for adipose tissue expansion, is associated with adverse metabolic outcomes, such as MASLD and severe IR.^551^, ^552^ However, adipose tissue fibrosis is still poorly understood in paediatric populations, largely due to the challenges involved in accessing tissue samples. Targeting adipose tissue fibrosis could potentially restore adipose tissue physiology and prevent the progression of obesity‐related complications. It remains unclear whether changes in adipose tissue occurring early in life persist into adulthood and contribute to the persistence of the obese phenotype, predisposing children to early cardiometabolic complications. One important biomarker of paediatric obesity is the ratio between VAT and total abdominal fat (VAT+SAT). A high VAT/(VAT+SAT) ratio has been associated with severe IR, increased risk of prediabetes, T2D, fatty liver and other cardiometabolic complications.^553^, ^554^ These changes are accompanied by the accumulation of DAGs and ceramides, which interfere with insulin signalling by inhibiting the proper phosphorylation of the insulin receptor. When IR occurs in adipose tissue, insulin fails to suppress adipose tissue lipolysis, resulting in an increased flux of FFA into the liver and muscles. The deposition of excess FFA in these organs is a major factor in the development of liver and muscle IR.^554^ In skeletal muscle, IR reduces glucose uptake, causing excess glucose to be redirected to the liver, where it is promptly converted into fat through a process called hepatic de novo lipogenesis (DNL).^554^, ^555^ This process contributes to hepatic IR and impairs the suppression of gluconeogenesis and glycolysis. DNL has been identified as a potential therapeutic target for managing T2D and dyslipidaemia, but it remains uncertain whether reducing DNL rates can be achieved without causing significant metabolic changes. The combined effect of increased FFA flux from adipose tissue and enhanced DNL leads to intrahepatic fat accumulation and MASLD, which is now the leading cause of liver transplant worldwide.^555^ In children, MASLD exacerbates IR, impairs insulin clearance, and is linked to beta‐cell deterioration, increasing the risk of progression to T2D.^556^, ^557^, ^558^ Puberty further worsens glucose metabolism, as adolescents experience physiological IR that peaks at Tanner stage 3 and returns to prepubertal levels by Tanner stage 5.^559^, ^560^ This variation is more pronounced in girls than in boys and is only partially explained by increased adiposity. The underlying mechanisms driving IR during puberty is unclear, but it may involve hyperinsulinaemia, which affects amino acid metabolism to facilitate rapid growth, as well as changes in growth hormone and IGF1 levels, which have been positively correlated with IR.^561^ Reduced peripheral glucose uptake, rather than increased hepatic gluconeogenesis, seems to be the primary driver of IR during puberty.^562^

Beyond fibrosis, oxidative stress emerges as a critical molecular mechanism in paediatric obesity. The imbalance between pro‐oxidant factors and antioxidant defences leads to increased production of reactive oxygen and nitrogen species. This oxidative milieu contributes to insulin resistance by impairing insulin signalling pathways and perpetuates a pro‐inflammatory state within adipose tissue. Moreover, oxidative stress‐induced lipid peroxidation exacerbates dyslipidaemia, further elevating cardiovascular risk. Understanding the interplay between oxidative stress and these metabolic disturbances is crucial for developing comprehensive therapeutic strategies aimed at restoring metabolic homeostasis in children with obesity.^563^, ^564^

### 11.3 Knowledge Gaps

Despite substantial progress, significant knowledge gaps remain regarding the impact of obesity on adipose tissue biology, particularly in paediatric populations. The exact characteristics of macrophages involved in inflamed adipose tissue remain poorly understood. Although many macrophages are thought to be M1‐type, emerging evidence points towards a unique population of metabolically activated macrophages whose role in disease progression has not been fully elucidated.^546^ Additionally, the phenomenon of adipose tissue fibrosis in children is not well understood, partly because of challenges in obtaining tissue samples. More research is needed to determine the extent to which fibrosis influences disease progression in paediatric populations.^548^, ^549^, ^550^ It is also not yet known whether the changes that occur in adipose tissue early in life persist into adulthood and how they may contribute to chronic obesity and related complications. Another important gap lies in understanding the long‐term effects of puberty‐induced IR on metabolic health. Puberty introduces physiological changes that lead to transient IR, but it remains uncertain how these changes influence long‐term cardiometabolic risk, particularly in children living with obesity.^559^ Furthermore, while there has been progress in treatment options, including pharmacological interventions like GLP‐1 receptor agonists and bariatric surgery, the long‐term efficacy and safety of these treatments in adolescents are not well established. More research is needed to assess their outcomes, particularly in relation to weight maintenance and metabolic health.^565^

Moving forward from individual biological mechanisms, environmental factors, particularly neighbourhood characteristics, play a crucial role in the development and progression of paediatric obesity and its metabolic complications. Children residing in neighbourhoods with high poverty and crime rates face increased risks of elevated BMI, partly due to limited access to recreational facilities and healthy food options. The presence of food deserts further exacerbates this issue, as the scarcity of affordable, nutritious food leads to unhealthy dietary patterns. Understanding the interplay between environmental factors and biological mechanisms is essential for developing comprehensive strategies to prevent and manage obesity and its metabolic consequences in paediatric populations.^566^

### 11.4 Implementation Strategies and potential issues

Addressing these knowledge gaps requires a multifaceted approach. Longitudinal studies are needed to determine whether early‐life changes in adipose tissue persist into adulthood and contribute to chronic obesity and metabolic complications. Such studies could help clarify the role of early adipose tissue modifications in the development of long‐term health issues. Additionally, improving our understanding of macrophage populations within adipose tissue through advanced imaging techniques and molecular analyses could shed light on their role in driving inflammation and metabolic dysfunction.^546^ By characterizing these macrophages in more detail, researchers could identify new therapeutic targets aimed at modulating inflammation. Efforts should also be made to develop non‐invasive diagnostic methods to identify adipose tissue fibrosis in children. Imaging biomarkers could help in detecting fibrosis at an early stage, which would facilitate timely intervention and enhance understanding of how fibrosis contributes to the progression of metabolic dysfunction.^550^ Investigating the mechanisms underlying puberty‐induced IR is also crucial. Understanding how pubertal changes contribute to cardiometabolic health will enable the design of targeted interventions to address these challenges in adolescents. Finally, standardized protocols for evaluating the long‐term efficacy of pharmacological and surgical treatments for paediatric obesity are essential. These protocols should include comprehensive definitions of comorbidities and detailed follow‐up data to assess treatment success and safety. Implementing these protocols will provide robust data on the effectiveness of treatments such as GLP‐1 receptor agonists and bariatric surgery, thereby guiding clinical decision‐making.^565^ Implementing these strategies comes with several potential challenges. Obtaining adipose tissue samples from children for advanced imaging and biopsy analyses presents ethical and practical challenges. These limitations hinder the ability to fully characterize macrophage populations and understand the development of adipose tissue fibrosis. Additionally, non‐invasive diagnostic tools for detecting adipose tissue fibrosis may not be as accurate as invasive biopsies, potentially delaying the identification of fibrosis until more severe symptoms emerge. This delay could reduce the effectiveness of early interventions aimed at preventing disease progression. Adherence to long‐term pharmacological treatments, such as GLP‐1 receptor agonists, is another challenge. These treatments often come with side effects, and the need for long‐term or lifelong use can be a significant burden on patients and their families. This issue highlights the importance of combining pharmacological treatments with lifestyle interventions to enhance adherence and outcomes. The cost of imaging techniques required to characterize fat depots accurately is also a limiting factor. High costs may restrict access to these diagnostic tools, limiting their use in routine clinical practice and ultimately affecting the ability to monitor disease progression and treatment effectiveness.

Access to bariatric surgery for adolescents is restricted not only by high costs but also by concerns about its perceived invasiveness and the lack of long‐term outcome data. These concerns include the potential for nutritional deficiencies and the psychological impact of undergoing major surgery at a young age. Addressing these challenges requires comprehensive education for patients and families about the risks and benefits of bariatric surgery, as well as careful post‐surgery monitoring of patients to ensure long‐term safety and effectiveness.

## 12 TASK 5. POINT TO POINT SUMMARY

**Table:** 

Knowledge gaps	Characteristics of macrophages in inflamed adipose tissue are unclear Adipose tissue fibrosis in children is not well understood, with challenges in tissue access limiting research Long‐term effects of puberty‐induced IR and treatment outcomes in adolescents are unknown
Implementation strategies	Conduct longitudinal studies to understand the persistence of early‐life changes in adipose tissue Develop non‐invasive methods to detect fibrosis early and improve understanding of pubertal IR mechanisms Establish standardized protocols for evaluating long‐term treatment efficacy for paediatric obesity interventions
Potential issues	Ethical and practical challenges in obtaining tissue samples limit understanding of macrophages and fibrosis Adherence to long‐term pharmacological treatments and cost of advanced diagnostics are significant barriers High cost and perceived invasiveness of bariatric surgery, alongside lack of long‐term outcome data, limit its accessibility

Abbreviation: IR, insulin resistance.

## 13 TASK 6. MANAGEMENT OF OBESITIES: CLINICAL NEEDS AND CURRENT GAP

### 13.1 Background

The management of obesity is complex and often requires a combination of lifestyle interventions, cognitive‐behavioural therapy and, in some cases, pharmacotherapy and bariatric surgery (BS) making personalized treatment approaches necessary.^567^, ^568^ Moreover, the variety and complexity of the different types of alterations should be contemplated, thereby making it necessary to address the management of ‘obesities’, in plural.^569^, ^570^

Recommended dietary guidelines have changed over the years according to different trends. Initially, very low‐calorie diets (VLCD) or low‐fat diets were routinely recommended for PlwO. While currently the focus is mainly placed on the overall eating pattern and not so much on specific food items or restrictive approaches, reaching a consensus about the optimal distribution of macronutrients for managing PlwO is proving difficult.^571^ Likewise, research and recommendations in the field of the specific type of exercise as well as indications on the total amount of physical activity have been frequently ignored or have not been developed at the required level.^572^

The use of obesity management medications (OMM) to assist in weight loss, is generally considered when lifestyle modifications alone have been insufficient and when the patient has a BMI of 30.0 kg/m^2^ or higher, or from 27.0 kg/m^2^ onwards with obesity‐related comorbidities.^573^ OMM works through various mechanisms that can result in a reduction of body weight. These mechanisms include appetite suppression, increased satiety, decreased absorption of dietary fats and increased energy expenditure at the same time as exerting a beneficial impact on obesity‐associated comorbidities.^574^, ^575^

Several endoscopic alternatives for weight loss have been developed and proven to induce weight loss but due to the relatively small studies and lack of long follow‐up data they will not be considered in detail in the present review. BS, on the contrary, has extensive evidence to show the best long‐term effects in adequately selected candidates with proven weight loss and resolution or at least improvement in most if not all associated comorbid conditions.^567^

### 13.2 Summary of the evidence

#### 13.2.1 Dietary management

Weight loss approaches commonly focus on decreasing intake of total fat since lipids encompass more calories than proteins or carbohydrates.^576^ Diets low in fat are reportedly associated with slightly less weight loss than those low in carbohydrates.^577^ However, low‐fat diets generally achieve better long‐term effects than the usual diet, depending on specific caloric restriction and frequency of monitoring. While high‐intensity dietary interventions with VLCD (<800 kcal per day) do achieve higher average weight losses than the conventional hypocaloric diet,^578^ the detrimental effects on whole‐body lean mass loss, thigh muscle area and total hip bone mineral reduction have to be considered.^579^ Thus, these types of diets are recommended by professionals only in patients in need of rapid weight loss or before surgery in patients with elevated waist circumference and marked steatotic liver disease.^580^ Ranking of foods based on their impact on the determination of circulating blood glucose excursions is referred to as the glycaemic index (GI). To that end, the glucose absorption of 50 g is used to compare the effect of specific foods to elevate glycaemia.^581^ Thus, foods with a high‐GI translate into rapid spikes in circulating glucose and insulin concentrations. Therefore, low GI diets are associated with an improvement in glycaemic variability, inflammation parameters and endothelial function in PlwO.^582^ In RCTs, low carbohydrate diets (i.e. below the 45% of total daily energy set by classic guidelines as the lower level for this macronutrient^583^ and maintaining a protein intake around .8–1.5 g/kg of ideal body weight in order to preserve fat‐free mass) are commonly used to reduce body weight as well as for T2D management.^584^ Protein and fats enhance satiety at the same time as producing a lower hypoglycaemia concomitantly, further decreasing hunger and overall food intake that produces a caloric deficit. Another hypothesis argues that diets low in carbohydrates can induce an approximately 200–300 kilocalorie increased metabolic rate than diets high in carbohydrates in isocaloric conditions.^585^ Low‐carbohydrate diets achieve a weight loss over 12–24 months similar to or slightly greater than low‐fat diets^586^, ^587^ and associated with an improvement in the dyslipidaemic profile (i.e. decreasing triacylglycerols, increasing HDL and changing LDL particle size) in people with or without T2D.^588^ However, in a meta‐analysis of 19 studies of low‐carbohydrate diets compared with isocaloric conventional ones, little or no difference in weight loss at 3–6 months nor at 12–24 months was found.^589^, ^590^

High‐protein diets are on the rise for weight loss due to the supposedly decreased adiposity and improved satiety. However, decades ago this approach was established through the Atkins diet, which was characterized by a diet high in proteins and fat (mainly of animal origin) and low in carbohydrates without restriction in total energy supporting that the diet‐induced thermogenesis or thermic effect of food, is augmented in high‐protein approaches.^591^ Interestingly, the secretion of gut neuropeptides that induce satiation, like GLP‐1 and glucose‐dependent insulinotropic polypeptide (GIP), is increased in diets high in protein content.^592^ Noteworthy, preservation of the fat‐free mass with high‐protein diets for weight loss is superior to standard hypocaloric, low‐fat or normoprotein diets. Thus hypocaloric, low‐fat hyperproteic diets may be associated with a more favourable profile with greater reductions in weight, fat mass and triglycerides, and a greater conservation of fat‐free mass and basal metabolic rate in the short term (12 weeks).^593^ However, with ketogenic diets (KDs), high in protein with a carbohydrate intake <10% (or <20–50 g/day), ketosis can take place. A profound decrease in the consumption of carbohydrates (<50 g/day) characterizes KDs along the parallel proportional elevation of protein and fat intake. A further increase in metabolic efficiency related to fat intake and an increase in the thermic effect of KDs may be achieved following the blunted appetite and elevated lipolysis.^594^ Other diets restricting carbohydrates in variable amounts exist either without dietary protein and fat restriction (such as the Atkins diet) or allowing a moderate intake of them.

In the presence of the small carbohydrate intake of KDs, the produced ketones in the liver are employed as the alternative source of energy. The usefulness of KDs in refractory epilepsy as well as potential beneficial effects against neurodegenerative diseases and cancer have been also reported. Nonetheless, the beneficial health‐related effects of KDs probably relate to more aspects, likely depending on a variety of multiple complex factors, one of which pertains to pro‐ or anti‐ inflammatory influences.^595^ While β‐hydroxybutyrate, might exert anti‐inflammatory properties,^596^, ^597^ KDs have been shown to be pro‐inflammatory leading to organ damage, affecting kidney, cardiac and bone health, among others.^598^, ^599^

Numerous health benefits pertaining to a lower mortality and cardiovascular disease incidence are reportedly related to the Mediterranean diet (MD).^600^, ^601^, ^602^ The healthy dietary pattern associated to the MD, includes a high intake of legumes, vegetables, cereals nuts and fruits, on the one hand, at the same time as a limited intake of processed foods, meat, cold meat products and dairy (except long‐preservable cheeses).^603^ While intake of alcohol within the traditional MD takes place, it is performed always during meals and in moderate amounts. Despite a potentially high total fat intake, it is based on a favourable monounsaturated to detrimental saturated lipids ratio, linked to the use of olive oil. MD is associated with improvements in fat mass, waist circumference, insulin sensitivity and glycaemic control in patients with or without T2D as well as in inflammation and reduction in LDL cholesterol. Initially outlined to decrease blood pressure without medication,^604^ the Dietary Approaches to Stop Hypertension (DASH) diet, is currently regarded as one of the most favourable eating patterns^605^ limiting the intake of sodium, favouring intake of multiple vegetables and fruits, while emphasizing the consumption of whole grains. In PlwO an effective decrease in body weight together with a reduction in insulin resistance, triacylglycerols, inflammatory markers and steatotic liver disease with the DASH diet has been observed.^606^

Currently, approaches that refer to the importance of taking care of the planet and sustainability are spreading among the population, developing variants of the plant‐based and vegetarian dietary patterns like the flexitarian, pescetarian, ovolactovegetarian, lactovegetarian, ovovegetarian and vegan approaches.^607^ The lacto‐ovovegetarian diet has been associated in cross‐sectional and cohort studies with lower body weight and a decreased incidence in cancer and cardiovascular events.^608^ When compared in the short term with a MD, both diets were equally effective in BMI and fat mass reduction. Nonetheless, vegetarian diets were more effective in decreasing LDL cholesterol concentrations, as opposed to the MD that exhibited a higher lowering of triacylglycerol levels.^609^ With vegetarian and vegan diets particular attention to potential iron and vitamin B12 deficiencies should be considered.

Specific periods of extremely reduced or no calorie intake at all characterize intermittent fasting (IF) approaches. Regimes can vary though the daily time‐restricted IF (16–18 h fasting per day), the alternate‐day fasting and the 5:2 IF (with intake of 900–1000 calories for 2 days each week) represent the most frequently applied. Some studies in people with T2D or metabolic syndrome show efficacy in weight loss compared to normal and low carbohydrate content, improvement in fat mass and waist circumference as well as in glycaemic control. Also, it is associated with a reduction in LDL cholesterol and postprandial lipemia in PlwO, improvement in the components of the metabolic syndrome (increase in HDL cholesterol, decrease in triglycerides, decrease in arterial hypertension) and an improvement in insulin sensitivity, inflammation (C reactive protein, IL‐6, IL‐18, TNF‐α) and endothelial function.^610^ Hypotheses support that time slots of limited dietary energy intake are sufficient to deplete hepatic glycogen stores that set off a switch in metabolism towards fatty acid and ketone use. Combination of the IF regimen with regular exercise reportedly translates into multiple long‐term improvements in physical and mental performance together with higher resistance to disease.^611^ Although several studies published in the last years confirm that IF can be useful for management of obesity, no superiority to conventional caloric restriction diets have emerged.^612^ More recently, attention has been drawn to the increased cardiovascular death association to an 8 h‐eating window in a national survey with 20,000 US participants.^613^ It is also important to remember that dietary interventions should not only focus on creating an energy deficit. Diets must be balanced in macronutrients, reduced in ultra‐processed foods and tailored to nutritional needs, clinical presentation and individual preferences.^614^ A challenge with interpreting results on specific diets is the fact that their literature is likely to suffer from generalizability biases^615^ as well as selective reporting and other publication biases. Most of the RCTs are short‐term and do not examine hard outcomes and many trials are not registered.^616^ Epidemiological associations of specific diets with favourable outcomes are even more subject to bias.^617^, ^618^

#### 13.2.2 Exercise

Comprehensive management of obesity should include physical activity as an essential element of every programme.^572^ Among the broad dimension of physical activity, exercise training, in particular aerobic training, is associated with an important though modest (approximately 2–3 kg) of extra weight loss in PlwO as compared to controls without training. Total loss of fat yields similar outcomes. Interestingly, a decrease in visceral adiposity with its associated cardiometabolic health benefit also takes place. Furthermore, regular physical activity exerts potent anti‐inflammatory effects, which are particularly relevant in obesity, where chronic low‐grade inflammation is a key driver of metabolic dysfunction. Exercise reduces the levels of pro‐inflammatory biomarkers such as C‐reactive protein (CRP), interleukin‐6 (IL‐6) and tumour necrosis factor‐alpha (TNF‐α), which are associated with insulin resistance and cardiovascular diseases. Additionally, physical activity enhances the production of anti‐inflammatory cytokines, such as IL‐10 and adiponectin, which help restore metabolic balance and insulin sensitivity.^619^, ^620^ In terms of cardiovascular health, studies have shown that aerobic and resistance exercise reduce blood pressure, improve endothelial function and enhance autonomic control of the heart, reducing the risk of cardiovascular events.^621^ These benefits are independent of weight loss, further reinforcing the importance of exercise beyond caloric expenditure.

However, data regarding the effect of exercise in maintenance of weight is currently not clear, though retrospective studies show the impact of relatively high‐volume exercise. It is important to emphasize that physical activity encompasses a broader range of movements beyond structured exercise, including activities of daily living such as walking, climbing stairs, household chores and occupational activities. International guidelines recommend at least 200–300 min of moderate‐intensity physical activity weekly.^622^ Regarding cardiovascular benefits, various exercise training modalities, including aerobic, resistance and concurrent training, have been shown to contribute differently to blood pressure reduction and control.^623^ Moreover, regular physical exercise is known to promote a favourable cardiovascular state by improving endothelial function through several mechanisms. Additionally, studies suggest that isometric exercise training may produce significant reductions in blood pressure, with mean reductions of between 10 and 13 mm Hg systolic, and 6 and 8 mm Hg diastolic.^624^ However, achieving these goals is often difficult, so it is also beneficial to reduce a sedentary lifestyle and incorporate physical activity in the form of active breaks or exercise snacks to reduce the risk of morbidity and mortality. For the preservation of the lean mass during weight loss resistance training is particularly recommended.^625^ While the somewhat restricted impact of structured exercise on weight loss should not be dismissed, a focus on increasing overall physical activity levels, even in small increments, leads to relevant health benefits such as improved cardiorespiratory fitness, reductions in visceral adiposity and enhanced metabolic health.^626^ Importantly, adherence to increasing physical activity in the long term still represents a huge challenge in any management strategy.^5^

#### 13.2.3 Cognitive‐Behavioural Management

Cognitive‐behavioural interventions can help PlwO to manage situations, behaviours and thoughts that contribute to or maintain weight gain and stress (including weight bias internalization) through several strategies (e.g. self‐monitoring, goal setting, problem solving, cognitive restructuring, stimulus control). Multicomponent psychological therapies are evidence‐based treatment options for obesity and weight management and represent the third key pillar of obesity management.^627^ Key findings from RCTs including integrated diet, exercise, and behavioural therapy—also known as behavioural treatment, comprehensive lifestyle modification or intensive lifestyle intervention—result in baseline body weight reductions of 5%–10% on average between 6 and 12 months, improved participants' levels of physical activity and food intake.^628^ These losses have been shown to improve other obesity‐related problems and lower the risk of T2D in people with impaired glucose tolerance. These advantages have also been linked to lower medical expenses, and there is no indication that the intensity of the intervention affected the effect, in contrast to the results for intermediate outcomes. Moreover, these interventions positively reinforce health behaviours (e.g. nutritional, medical therapy, physical activity, adherence to follow‐up and treatment). In addition, they will generate a deeper understanding of the underlying reasons and conditions of the disease (e.g. thoughts, attitudes, emotions, expectations and barriers).^628^, ^629^

#### 13.2.4 Obesity management medications

Long‐term weight maintenance is challenging, and weight regain is common.^630^ The addition of pharmacotherapy for overweight and obesity increases the proportion of people achieving more significant weight loss success and weight maintenance. Additional weight reduction with pharmacotherapy is 5%–15%, which conveys multiple metabolic, cardiovascular and mechanical benefits and improved quality of life.^631^, ^632^

Orlistat is a pharmacological agent used in the treatment of obesity, approved for both prescription and over‐the‐counter use. Its primary mechanism of action is the inhibition of dietary fat absorption, which helps to reduce caloric intake and promotes weight loss.^633^ Orlistat was approved by the EMA and the FDA in 1998 and 1999, respectively, and remains a sometimes‐used medication for managing obesity due to its efficacy and low side effects.^567^, ^634^, ^635^ Orlistat functions by inhibiting gastric and pancreatic lipases, enzymes that play a crucial role in dietary fat digestion. Orlistat prevents the breakdown of triacylglycerols into absorbable free fatty acids and monoacylglycerols by binding them to these lipases. Consequently, approximately 30% of the ingested fat is excreted undigested in the faeces. This reduction in fat absorption translates into a caloric deficit, contributing to the loss of total body weight. Clinical trials have demonstrated that orlistat, when combined with a reduced‐calorie diet, leads to significant weight loss when compared to using only diet. On average, patients taking orlistat can expect to lose about 5%–10% of their initial body weight over 6 months to a year. This weight reduction is clinically meaningful, as even a modest weight loss of 5%–10% can improve obesity‐related comorbidities, such as T2D, hypertension, and dyslipidaemia.^636^, ^637^ In addition to weight loss, orlistat has been shown to improve other health parameters. Studies indicate that orlistat can reduce levels of LDL cholesterol, improve glycaemic control in people with T2D, and lower blood pressure.^638^ These benefits make orlistat a valuable component of a comprehensive obesity management plan.

The combination of phentermine and topiramate has been increasingly recognized for its efficacy in the pharmacotherapy of obesity. Its use was approved by the FDA in 2012, while it was refused by the EMA in 2013 by concerns about the long‐term effects of phentermine on the heart and blood vessels, and the long‐term psychiatric effects related to topiramate. Phentermine is a sympathomimetic amine, functioning as an appetite suppressant. It stimulates norepinephrine release in the hypothalamus, leading to reduced hunger sensations. This mechanism is somewhat like to that of amphetamines but is specifically targeted at appetite control without inducing the broader range of stimulant effects seen with amphetamines. Topiramate, on the other hand, is an anticonvulsant medication that has been found to promote weight loss through several mechanisms. It enhances gamma‐aminobutyric acid action, inhibits carbonic anhydrase and modulates voltage‐gated ion channels. These actions contribute to a decrease in appetite and an increase in feelings of fullness, although the exact pathways through which topiramate aids in weight loss are not entirely understood.^639^ One of the pivotal studies leading to the approval was the CONQUER study, a randomized, double‐blind, placebo‐controlled trial involving 2487 participants. The trial evaluated the efficacy and safety of phentermine/topiramate over a year. Participants who received the highest dose (phentermine 15 mg/topiramate 92 mg) achieved a total body weight loss (TBWL) on average of 9.8%, as opposed to a 1.2% decrease in the control placebo group. Furthermore, substantial improvements were observed in cardiovascular risk factors, including reductions in blood pressure, triglycerides, and fasting blood glucose levels.^640^ Another significant study, the EQUIP trial, focused on people with a BMI ≥35 kg/m^2^. The results mirrored those of the CONQUER trial, with the high‐dose group experiencing a 10.9% weight loss when compared to the 1.6% loss in the control group on placebo.^641^ Additionally, a 2‐year extension study, SEQUEL, confirmed that the weight loss achieved with phentermine/topiramate was maintained over a longer period, with sustained improvements in metabolic parameters.^642^

The combination bupropion/naltrexone leverages the unique mechanisms of two established medications—bupropion, an antidepressant and smoking cessation aid, and naltrexone, an opioid antagonist—to promote weight loss by influencing central nervous system (CNS) appetite control and energy homeostasis. Bupropion is primarily known as a norepinephrine‐dopamine reuptake inhibitor. By enhancing dopaminergic and noradrenergic activity, bupropion can decrease food intake and augment expenditure, thereby promoting weight loss. Naltrexone exploits antagonism of the opioid receptor, usually applied to manage alcohol and opioid dependence. In the context of weight management, naltrexone targets the CNS, particularly the hypothalamus and mesolimbic dopamine system, which are involved in controlling food intake and reward. Naltrexone is believed to modulate the pro‐opiomelanocortin (POMC) neurons, which exert a pivotal role in decreasing appetite and increasing energy expenditure. By blocking opioid receptors, naltrexone prevents the inhibition of POMC neurons, thus promoting satiety and reducing cravings.^643^ Clinical trials have demonstrated that the bupropion/naltrexone combination is effective in promoting weight loss in PlwO. On average, patients treated with this combination can achieve a weight loss of approximately 5%–10% of their initial body weight over a period of 1 year. This level of weight loss is clinically significant and can lead to improvements in various obesity‐related conditions, such as T2D, hypertension and dyslipidaemia.^644^, ^645^

**FIGURE 4:**
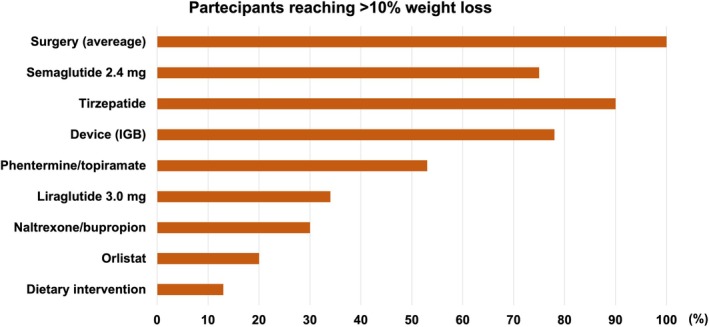
A proportion of participants achieving 10% or more total body weight loss after 1 year with currently available therapeutic approaches (based on data reviewed in Perdomo et al.^567^).

The profound impact on body weight and metabolic health have positioned GLP‐1 receptor agonists and their combinations as potentially transformative management options for obesity by safely and effectively closing the gap with the weight loss percentages usually achieved by BS (Figure 4). Liraglutide is an effective drug for the treatment of obesity. Originally developed for diabetes treatment, liraglutide's ability to promote weight loss led to its approval for obesity management by the FDA in 2014 and by the EMA in 2015. This medication is particularly notable for its dual benefits in both glycaemic control and weight reduction. Liraglutide is an agonist of the GLP‐1 receptor. GLP‐1 is an incretin hormone naturally secreted in the gut after the intake of food playing a crucial part in appetite control and intake of food, slowing the emptying of the stomach, promoting satiety, and stimulating insulin secretion. Liraglutide resembles the effects of GLP‐1, via receptor binding and activation, which results in reduced appetite and food intake, thereby contributing to weight loss. Additionally, liraglutide's impact on the CNS involves modulation of appetite‐regulating centres in the brain, particularly the hypothalamus.^646^ This central action helps patients feel fuller sooner and reduces cravings, making it easier to adhere to a reduced‐calorie diet.^567^, ^635^ Clinical trials in PlwO have evidenced liraglutide's effectiveness in achieving significant weight reduction. Patients treated with liraglutide achieved a 5%–10% weight loss on average from baseline over 1 year, which is clinically significant. Such weight decrease is related to amelioration in obesity‐associated conditions like T2D, hypertension, dyslipidaemia and cardiovascular disease risk factors. For instance, the SCALE (Satiety and Clinical Adiposity—Liraglutide Evidence) trial showed that 3.0 mg of liraglutide led to an average loss of weight of 8.0% compared to 2.6% in the control placebo group after 56 weeks.^647^ Furthermore, improvement in the glycaemic control following liraglutide treatment was shown to, making it particularly beneficial for PlwO and T2D.^648^

Semaglutide is another GLP‐1 receptor agonist that has shown substantial efficacy in the treatment of obesity.^649^ Initially developed for managing T2D, semaglutide has been repurposed for body weight reduction, with FDA and EMA approval granted for this indication in 2021. Its ability to promote significant weight loss has made it a valuable addition to obesity management strategies. Semaglutide resembles the endogenous hormone GLP‐1, which is implicated in appetite regulation and insulin secretion, leading to a decrease in overall caloric intake and slowing gastric emptying, which prolongs the feeling of fullness after meals.^567^, ^635^ Several large‐scale RCTs have documented semaglutide's efficacy in body weight loss.^650^ The STEP (Semaglutide Treatment Effect in People with obesity) programme, which encompasses multiple Phase 3 trials, has provided robust evidence of its effectiveness. In these studies, participants receiving semaglutide 2.4 mg once weekly, in combination with lifestyle interventions, achieved an average weight loss of approximately 15%–20% of baseline body weight after 68 weeks.^651^ This level of weight reduction is significantly higher compared to other currently available OMM. Such significant weight loss is associated with improvements in various obesity‐related conditions, including T2D, hypertension, dyslipidaemia, steatotic liver disease, heart failure, osteoarthritis, sleep apnoea and can lead to an overall enhancement in quality of life.^652^, ^653^, ^654^, ^655^, ^656^, ^657^

Tirzepatide uses the dual agonism of GLP‐1 and GIP receptors as a novel approach.^567^, ^635^ It was approved for obesity management by the FDA and EMA in 2023. By simultaneously activating these two incretin receptors, tirzepatide harnesses the synergistic effects of both hormones. GIP also plays a role in enhancing insulin secretion but has additional effects on adipose tissue, which may contribute to improved fat metabolism and further appetite suppression. This dual mechanism makes tirzepatide distinct from other GLP‐1 receptor agonists. By engaging both GLP‐1 and GIP pathways, tirzepatide enhances weight loss and metabolic benefits more effectively than targeting the GLP‐1 receptor alone.^658^ The efficacy of tirzepatide in treating obesity has been demonstrated through robust clinical trials. For example, the SURMOUNT‐1 trial focused on PlwO without T2D, where participants experienced a weight loss of 20.9% on average over 72 weeks, marking one of the most significant weight reductions observed with pharmacotherapy to date.^659^ The SURPASS‐2 trial evaluated tirzepatide's weight loss efficacy in people with T2D and demonstrated an average weight reduction of 8.5%–13.1% of initial body weight, depending on the dose, over a 40‐week period.^660^ Such significant weight loss was beneficial for improving obesity‐related comorbidities, including cardiovascular risk factors, T2D, hypertension, dyslipidaemia, metabolic dysfunction‐associated steatohepatitis with liver fibrosis and sleep apnoea.^661^, ^662^, ^663^

#### 13.2.5 Endoscopic procedures and bariatric surgery

To fill the gap between OMM and BS endoscopic bariatric procedures (EBP) were developed for PlwO.^664^ People exhibiting a BMI ≥27.5 kg/m^2^ who are not candidates for surgery for clinical reasons or who prefer less invasive, non‐surgical approaches as well as patients with a BMI > 50 kg/m^2^ might benefit from an intragastric balloon (IGB) as a bridge for weight loss before surgery. Among the several available EBP, the IGB and the endoscopic gastroplasty (EG) are the currently most applied ones. Despite the initial encouraging results of EBP for obesity management, prospective RCTs with long‐term follow‐up are required to validate their true contribution and thus escape the focus of the present review.

A meta‐analysis of the scarce and small RCTs of non‐adjustable fluid‐filled IGB together with diet compared to lifestyle modification alone showed a modest BMI loss of 1.4 kg/m^2^ together with a 3.6 kg weight loss.^665^ Subsequently, a TBWL of 15.0% at 32 weeks with the adjustable fluid‐filled IGB was reported in an RCT versus a 3.3% loss with lifestyle alone.^666^ Although complications related to the devices are infrequently (≤1%) reported, the unforced hyperinflation of the balloon, obstruction, ulceration and perforation of the stomach can happen.^667^ The EG reduces the stomach size by plicating with sutures the greater curvature, thereby restricting the gastric volume. While TBWL achieved ranges from 10% to 16% in small retro‐ and prospective studies with short‐term follow‐ups, durability of outcomes has been disappointing.^668^ Complications affect around 1% of patients, encompassing inflammatory fluid collections or abscesses around the stomach, important bleeding and deep vein thrombosis. Overall, the lack of long‐term outcomes with robust methodology hampers the identification of the potential clinical application niche for EBP.

The beneficial and sustained effects on TBWL and comorbidity control of BS for adequately selected candidates is out of doubt showing so far, the best long‐term outcomes. In addition, BS can disrupt the progression to prediabetes or T2D and substantially improve other obesity‐associated diseases. Sleeve gastrectomy and the Roux‐en‐Y gastric bypass (RYGB) via the laparoscopic approach are the most frequently performed procedures.^669^ The one‐anastomosis gastric bypass (OAGB) together with the adjustable gastric banding and the biliopancreatic diversion represent the rest of the main operations performed. The RYGB reportedly exhibits better long‐term results in 5–7 years of follow‐up,^670^, ^671^ with an average 25% TBWL after a follow‐up of 20 years.^331^ Longer follow‐up studies are required to inform about the lasting effects of sleeve gastrectomy on weight loss and its clinical significance. While the OAGB may achieve similar or even better results it is bound to have higher nutritional deficiencies, and a high rate of alkaline reflux. BS is equally effective in improving or resolving glycaemic metabolism including cardiometabolic risk, dyslipidaemia, hypertension, steatotic liver disease, obstructive sleep apnoea, early‐stage diabetic chronic kidney disease, micro‐ and macrovascular events as well as mortality.^672^, ^673^, ^674^, ^675^

### 13.3 Knowledge gaps

Despite significant advances, several knowledge gaps remain to be addressed. Bridging these gaps will require a concerted effort from researchers, clinicians and policymakers to ensure that patients receive the best possible care. In general, determination of the adequate distribution of the different macronutrients to ensure weight loss at the expense of fat mass but also prevention of weight gain ranging from conservative lifestyle measures only up to the more interventional BS interventions is still a topic of debate. The same holds true for the physical activity prescription as regards type and frequency of exercise. Moreover, the marked variation in the inter‐individual response to weight loss is observed irrespective of the treatment approach, thereby highlighting the necessity to schedule appropriately individualized plans in view of specific characteristics such as eating behaviours, basal energy expenditure, body composition, clinical conditions and socio‐economic circumstances that could lead to better outcomes than the standard of care.^567^ The advent of nutrient‐stimulated hormone‐based drugs with GLP‐1 receptor agonists, their combinations and new compounds has been a real game‐changer with true transformative potential as well as a yet unidentified landscape that needs to be better analysed.

A relevant knowledge gap is the lack of personalized approaches in obesity management. The one‐size‐fits‐all approach in obesity treatment is increasingly being replaced by personalized medicine, which tailors interventions based on a person's genetic, metabolic and behavioural profile.^676^ Individual responses to treatment can vary widely due to genetic, metabolic and behavioural differences.^677^ Understanding the environmental and genetic influences that favour drug efficacy and adverse effects is crucial for developing personalized treatment plans and particularly so for pharmacotherapy and BS.^569^ Research into pharmacogenomics—how genes affect a person's response to drugs—could lead to more tailored and effective therapies.^678^ Thus, health professionals should evaluate all the intervening factors encompassing from genetic predisposition, to metabolic and endocrine disorders as well as psychological and sociocultural influences, together with the use of medications, that may be related to weight gain, in order to perform an extensive clinical evaluation to determine the various phenotypes of disease expression.^569^ Determining all these factors allows health professionals to prescribe more appropriate therapeutic strategies, ensuring that treatments are tailored to each patient and better results might be obtained in adherence and long‐term outcomes.^679^, ^680^, ^681^ However, this area is still in its early stages, and much more research is needed to translate these insights into clinical practice.

The long‐term efficacy and safety of OMM are not well established.^638^ Most clinical trials for these drugs are relatively short‐term, typically lasting one to 2 years, which is insufficient to fully understand the long‐term implications of these treatments.^682^, ^683^ Chronic administration of OMM could potentially lead to unforeseen adverse effects or diminished efficacy over time.^634^ Therefore, there is a need for long‐term studies that follow patients over several years to monitor the sustainability of weight loss, the emergence of side effects and the overall impact on health.^684^, ^685^

One major gap lies in the incomplete understanding of how OMM exerts their effects at the molecular and cellular levels. While some medications, such as orlistat, work by inhibiting fat absorption, others like GLP‐1 receptor agonists (e.g. liraglutide) and appetite suppressants (e.g. phentermine) target brain pathways involved in hunger and satiety. However, the precise mechanistic underpinnings for weight regulation of these agents have not been fully elucidated. For example, the long‐term effects of GLP‐1 receptor agonists on the CNS and their interaction with other metabolic pathways remain poorly understood.^686^ This knowledge gap hinders the optimization of existing drugs and the discovery of novel therapeutic targets.^687^

Combination therapies, which use two or more medications to target different mechanisms of obesity, have the potential to enhance treatment efficacy.^567^, ^688^, ^689^ However, there is limited knowledge about the optimal combinations of drugs and their potential interactions.^635^, ^643^ More research is needed to determine the best combinations that maximize weight loss while minimizing side effects.^690^

Obesity is often accompanied by comorbid conditions such as T2D, hypertension, and dyslipidaemia.^691^ While some OMM have been shown to improve these conditions, the extent to which different medications affect various comorbidities is not fully understood.^573^, ^692^ This gap in knowledge limits the ability to choose the most appropriate drug for each patient with specific comorbidities.^575^ Comprehensive studies examining the outcomes of obesity pharmacotherapy on a wide scope of comorbid alterations are essential to provide better clinical guidance.

While obesity pharmacotherapy is effective in inducing weight loss and stimulating metabolic health, its effects on body composition, particularly muscle and bone integrity, are not well understood.^693^ This lack of information is critical, as the preservation of muscle mass, function and skeletal health is vital for overall functionality, mobility and long‐term well‐being.^694^ More research is needed to comprehensively evaluate how OMM influence muscle and bone, ensuring that weight loss strategies do not inadvertently compromise these essential components of physical health.^695^

### 13.4 Implementation strategies and potential issues

Although the motivation to lose weight in PlwO is evident, opportunities for earlier intervention by healthcare professionals may be missed.^696^ Determining the best dietary‐nutritional management for obesity is one of the great current challenges. Gathered evidence that some combinations of proven dietary interventions provide the most benefit to improve specific comorbidities needs to be harnessed. In PlwO and prediabetes or insulin resistance or T2D, eating patterns such as the MD could be combined, preferably with the consumption of foods with a low glycaemic index, which can translate into earlier improvements in insulin resistance, allowing, therefore, greater weight losses at the expense of fat mass. Medical nutrition therapies proposed must be adapted to the clinical characteristics, physical activity and exercise as well as preferences of each patient given that the most important aspect of any intervention is long‐term adherence.^697^

The improved safety and efficacy of OMM represent a substantial therapeutic advancement, yet disparities in access to not only pharmacological treatments but also other management approaches persist. The cost of new‐generation OMM remains a major barrier, as many of these treatments are not covered by health insurance or national healthcare systems in several countries. Prior to starting pharmacotherapy, conducting nutritional education sessions with patients (e.g. ensuring appropriate macronutrient intake, dietary fibre, and hydration) could facilitate adherence and management of side effects, which are common reasons for medication discontinuation. The same is true for BS, which remains the most effective long‐term treatment for severe obesity in adequately selected patients, yet is often inaccessible due to high costs, limited healthcare provider availability, and strict eligibility criteria that may exclude many individuals who would benefit from it. Moreover, the outcome improvements achieved by the latest OMM calls for BS to also maximize its beneficial effects while reducing potential complications.

Effective management of obesity through pharmacotherapy involves more than just drug development; it requires robust implementation strategies to ensure that treatments are accessible, acceptable and beneficial to all patients, regardless of socio‐economic status. In fact, in order to achieve the greatest health advantages, it might be necessary to use the new OMM with behavioural treatment to achieve a greater weight loss and deal with the challenges of maintaining weight loss.^628^ However, access to behavioural therapy, psychological counselling and structured weight management programmes is often limited, particularly in underserved areas. These approaches encompass a range of activities that can enhance the effectiveness of pharmacotherapy and/or BS. By adopting these strategies, healthcare systems can enhance the utilization and effectiveness of OMM and BS, ultimately leading to better health outcomes for PlwO.

Ongoing research and innovation are essential to refine and improve implementation strategies for obesity pharmacotherapy.^575^, ^690^, ^698^ This includes studying the real‐world effectiveness of OMM,^699^, ^700^ exploring new delivery methods (such as long‐acting injectables) and developing personalized treatment approaches based on genetic and metabolic profiling.^698^, ^701^ Collaboration between researchers, clinicians and policymakers is essential to drive continuous improvement in obesity management and ensure that treatment advances reach all individuals in need, not just those with financial or geographic advantages.^698^, ^702^

A critical component of implementing therapy for obesity is educating PlwO about their treatment options and engaging them in their own care with a non‐stigmatizing approach.^703^, ^704^ This involves providing clear, comprehensive information about dietary and physical activity recommendations, stress and sleep management, health behaviours, how OMM and BS work, their potential side effects and the expected outcomes.^705^ Effective education can improve adherence to overall management in general as well as for specific therapeutic regimens and empower people to make informed decisions about their health.^706^, ^707^ Strategies such as personalized counselling, educational materials and digital health tools (e.g. mobile apps and online platforms) can facilitate better understanding and engagement.^708^, ^709^ However, adherence to obesity treatments does not occur in isolation, as individuals are constantly influenced by their surrounding obesogenic environment—a setting that promotes excessive calorie consumption, physical inactivity and barriers to healthy living.^710^, ^711^, ^712^, ^713^ Moreover, political implementation of some strategies could further reduce the impact of obesity and the need to design more and better interventions.^714^ Unhealthy food availability, limited access to recreational spaces, food marketing, socio‐economic constraints and cultural norms can all act as barriers, reducing the long‐term effectiveness of educational interventions. Therefore, in addition to individual‐level strategies, it is imperative to address environmental and policy‐level factors that contribute to obesity.

Healthcare providers exert a key role in the implementation of obesity management.^715^ However, many providers may lack the necessary time and training to effectively prescribe dietary approaches, exercise and OMM at the same time as appropriately decide about the integration of these therapeutic options together with BS. Most health professionals have not received education on managing overweight and obesity during their academic training, whether during undergraduate, specialty and/or postgraduate studies.^716^ Implementing comprehensive training programmes for healthcare professionals—including physicians, nurses, nutritionists, mental health providers and pharmacists—can enhance their ability to identify suitable candidates for each treatment alternative, manage side effects, and support long‐term weight management.^685^ Training should also cover the importance of a multidisciplinary approach and follow‐up.

Implementing a multidisciplinary approach to obesity treatment can significantly improve outcomes. Multidisciplinary care teams, comprising dietitians/nutritionists, exercise physiologists, psychologists and medical specialists, can provide comprehensive care that addresses the multifaceted nature of obesity.^568^, ^717^ Pharmacotherapy and BS are most effective when combined with lifestyle interventions such as diet, exercise and behavioural therapy.^685^ However, in practice, this integration is often lacking. PlwO may receive medication without adequate support for making lifestyle changes, which can undermine the effectiveness of pharmacotherapy. A comprehensive, multidisciplinary approach is needed to ensure that patients do actually receive the necessary support to make lasting changes that will have real impact on their health.^567^ Frequent communication and coordination among team members are fundamental to guarantee that PlwO receive holistic, individually tailored and continuous care.^718^

Access to healthy foods and nutrition, engagement in physical activity and exercise possibilities, at the same time as psychological interventions, OMM and BS can be major barriers for many PlwO.^719^ Strategies to improve access include advocating for public health proven approaches with full insurance coverage that reduce out‐of‐pocket costs. Policymakers and healthcare organizations should work to include healthy options and OMM in standardized protocols to develop pricing strategies that make these treatments affordable.^720^, ^721^ Additionally, telemedicine and remote care options may help reach PlwO in underserved areas, providing them with access to necessary management and follow‐up care.^722^, ^723^

Continuous monitoring and follow‐up are crucial for the success of obesity management.^567^ Regular follow‐up appointments allow healthcare providers to estimate the safety and efficacy of the care provided, make necessary adjustments and offer ongoing support and motivation to PlwO.^706^ Implementing structured follow‐up protocols and using health information technologies, such as patient portals and electronic health records, can enhance the process tracking and ensure timely interventions.^724^, ^725^

Unfortunately, one of the health system's relevant challenges is implementing a chronic care model. This highlights the great need for management at the first level of care to form multidisciplinary teams that have training in the management of disease. Continuous monitoring allows the interdisciplinary team to provide feedback to the patient about their behaviour and to make the necessary adjustments.^568^, ^726^ It is necessary to provide health professionals with easy and intuitive technology to record patient data in public and private systems so that the characteristics of the population can be analysed locally. In turn, these systems can be sent to a centralized database that allows for the obtaining of national health indicators and the evaluation of the results of the strategies implemented in the short, medium, and long term for the management of overweight and obesity.^709^

The application of lifestyle interventions, OMM and BS presents numerous challenges that can impact its effectiveness, accessibility and safety. On the one hand, the development of highly effective OMM and BS can lead to the false belief that it is not necessary to maintain a healthy lifestyle, which is fundamental to avoid the weight gain that takes place when stopping pharmacological treatments or after undergoing BS. Likewise, the lack of adherence to health behaviours, such as dietary and physical activity recommendations or stress management makes it difficult to identify which intervention might be the best option for the management of obesity in each scenario.^727^ Finally, acknowledgement of additional circumstances related to exposure to obesogenic factors that have been underestimated for many years, such as high environmental temperatures, intrauterine and intergenerational effects, epigenetics, microbiota, sleep reduction and endocrine disruptors, among others, makes their contemplation further necessary when considering the most successful management for PlwO.^728^

**FIGURE 5:**
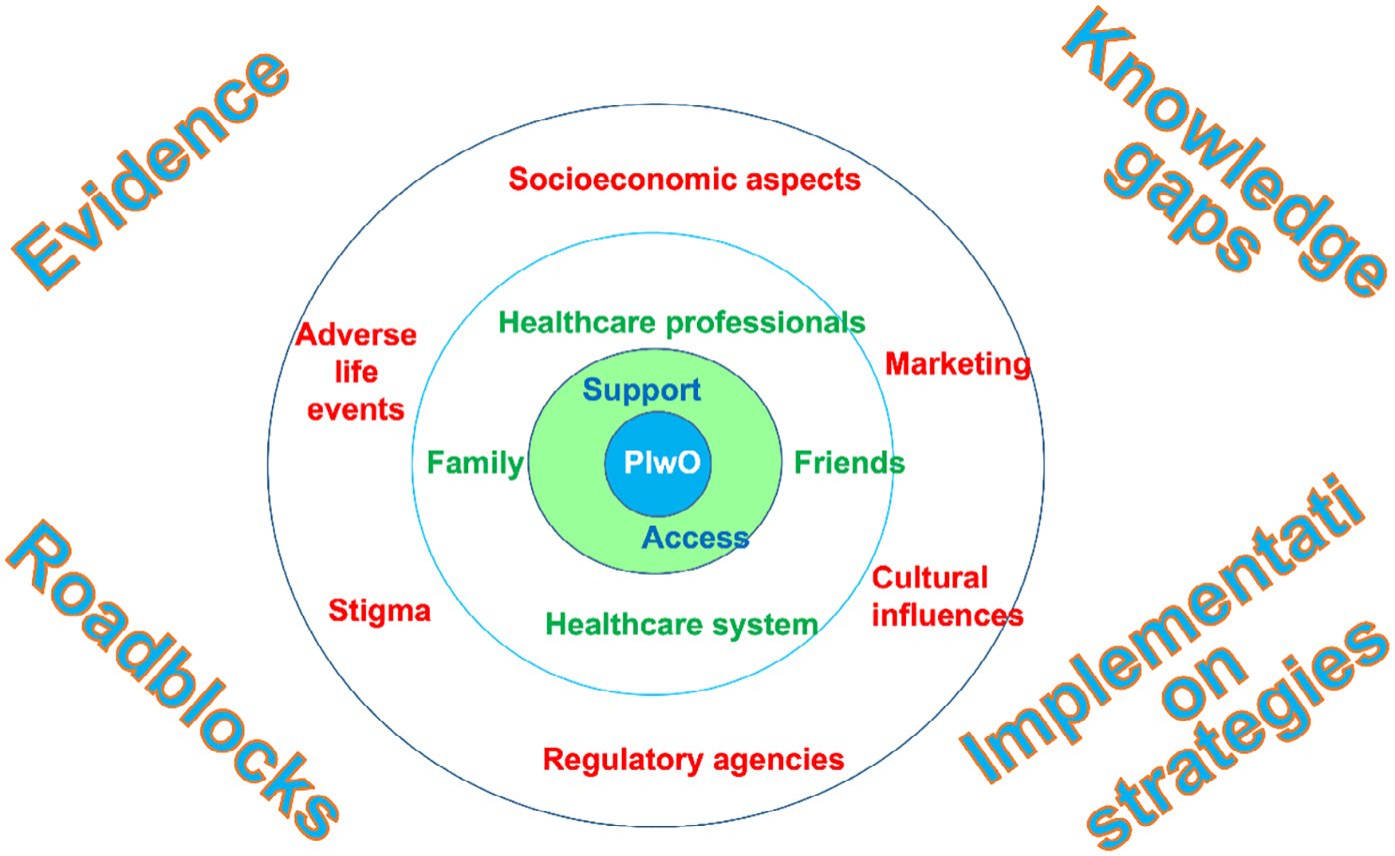
Overview of the elements that can influence and shape the care of people living with obesity (PlwO).

**FIGURE 6:**
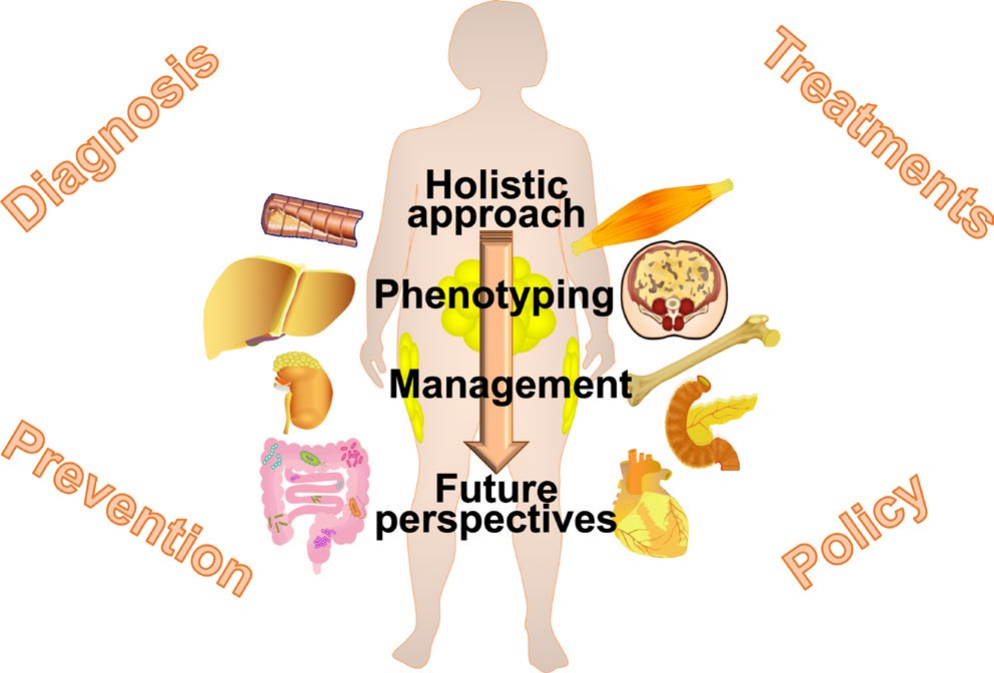
Schematic representation on how a holistic approach based on detailed phenotyping will improve an individualized diagnosis and treatments in the future of obesity management that need to translate into better prevention strategies and policy indications.

While multipronged approaches for obesity hold promise, their application is fraught with challenges that need to be carefully addressed to maximize benefits and minimize risks (Figure 5). By tackling these challenges through holistic approaches centred on the PlwO via comprehensive strategies, healthcare providers can improve the diagnosis and management of obesity, enhance health outcomes as well as prevention (Figure 6). One of the primary issues is the variable efficacy in response to the diverse treatment modalities among different persons.^567^ While some patients experience significant weight loss, others exhibit minimal effects. This variability can be attributed to genetic differences, metabolic factors and individual health conditions.^685^ The one‐size‐fits‐all approach often taken for obesity management does not account for these differences, leading to suboptimal outcomes for some patients. More research is needed to understand the factors influencing individual responses to better tailor treatments.^729^ Severely restrictive diets, OMM and BS can have notable side effects, ranging from mild gastrointestinal issues to nutritional deficiencies, severe cardiovascular and psychiatric effects, including increased suicidality.^730^ For example, drugs like orlistat can cause gastrointestinal discomfort, while others like phentermine can augment the risk of hypertension and cardiac disease.^634^ These side effects can seriously compromise overall health and need to be carefully considered when deciding about treatment options. Ensuring long‐term OMM safety is also a concern, as many clinical trials are short‐term, and the long‐term impact of these medications has not been fully explored.^573^

Adherence to lifestyle changes, OMM and post‐bariatric surgery regimens is a major challenge. Patients may discontinue therapeutic approaches due to side effects, lack of immediate results or the inconvenience of long‐term regular adherence. Behavioural factors and lack of motivation also play a role in non‐adherence.^731^ Without consistent follow‐up, the benefits of treatment alternatives can be significantly diminished. Strategies to improve adherence, such as patient education, support groups and adherence‐promoting technologies, are essential but often underutilized.^732^ The cost of healthy lifestyle choices, OMM or BS can be inaccessible for many PlwO, particularly those without adequate insurance coverage.^733^ High costs can limit access, especially among populations that are low in income and often at a higher risk for obesity. Additionally, insurance companies may be reluctant to cover some treatment options, viewing them as non‐essential or lifestyle drugs when obesity is not contemplated as a complex, chronic disease. Addressing the financial barriers through policy changes and advocating for better insurance coverage is crucial to improving access.^721^

The stigma associated with obesity can affect PlwO's willingness to seek and adhere to treatment.^685^ Many PlwO face bias and judgement, which can lead to feelings of shame and low self‐esteem. This stigma can deter PlwO from pursuing specialized care or cause them to abandon it prematurely. Healthcare providers must approach obesity management with sensitivity and specific support to mitigate these psychological barriers.^622^, ^685^ Regulatory challenges also pose issues especially in the application of OMM and BS. Pharmacological and surgical approaches for obesity management also raise concerns about medicalization, marketing and justice (since due to their high cost their application will disproportionately benefit the wealthy).^734^

## 14 TASK 6. POINT TO POINT SUMMARY

**Table:** 

Knowledge gaps	Identification of predictive factors for treatment success How to best articulate and apply personalized approaches in a multipronged management with different granularity of determinations Full mechanistic understanding of drug action and combination therapies is limited as is the impact on specific comorbidities Long‐term safety and efficacy of current obesity management medications and their combinations is lacking True impact of weight loss in an increasingly ageing population is uncertain Limited research on the economic impact of obesity and cost‐effectiveness of weight loss interventions Need for economic studies assessing the financial benefits of obesity reduction, including healthcare cost savings and improved workforce productivity
Implementation strategies	Opportunities for early diagnosis and intervention by healthcare professionals are missing Improve long‐term adherence to therapeutic approaches in a chronic relapsing disease Explore real‐world effectiveness and new methods of treatment delivery Engage and empower people living with obesity in care Improve and develop comprehensive training programmes for healthcare professionals of multidisciplinary teams Provide continuous monitoring and follow‐up Design and develop local and national monitoring systems of patient data Integrate cost‐effectiveness research into national obesity strategies to inform policymakers on resource allocation Encourage investment in prevention and treatment programmes by demonstrating long‐term economic benefits
Potential issues	Better understanding of the factors influencing individual responses is needed for tailoring of care Ensure the safety of treatment approaches and lack of detrimental effects associated with profound weight loss in vulnerable patients Address barriers to equitable access and cost of obesity management Avoid stigmatization of people living with obesity Demonstrating the economic viability of obesity treatment interventions may be challenging due to variations in healthcare infrastructure and funding models Need for interdisciplinary collaboration between economists, policymakers and healthcare professionals to optimize resource allocation

## 15 TASK 7. ENHANCING METHODOLOGICAL AND BIOSTATISTICAL RIGOUR IN OBESITY RESEARCH

### 15.1 Background

Obesity is a multifaceted public health issue with complex etiologies encompassing lifestyle, genetic, and environmental factors. While numerous studies aim to address obesity prevention and treatment, methodological and biostatistical shortcomings often limit the interpretability and applicability of their findings. This critique examines prevalent issues within current research paradigms.

### 15.2 Summary of the evidence

#### 15.2.1 Methodological Limitations

##### Study design inadequacies

Many studies employ cross‐sectional designs that are incapable of establishing causality. Longitudinal designs are less common but are crucial for understanding the temporal relationship between interventions and outcomes. Contrariwise, randomized Controlled Trials (RCTs) are the gold standard for evaluating interventions, but they are often logistically challenging and expensive in obesity research, leading to a reliance on less rigorous quasi‐experimental designs.

##### Population and sampling concerns

Selection bias is a recurrent issue due to non‐randomized sampling methods. This can result in study populations that do not accurately represent the broader community. In addition, small sample sizes and inadequate power calculations compromise the generalizability of results and the ability to detect significant differences or changes over time.

##### Intervention challenges

Heterogeneity in intervention components, duration, and intensity makes it difficult to compare results across studies. However, poor adherence to interventions and high dropout rates can lead to attrition bias, where the characteristics of those who complete the study differ from those who do not.

##### Outcome measures and follow‐up

While reliance on self‐reported measures for dietary intake and physical activity is prone to reporting bias and inaccuracies, insufficient follow‐up durations fail to capture long‐term outcomes and sustainability of interventions.

### 15.3 Implementation strategies and potential issues

#### 15.3.1 Biostatistical issues

While overreliance on *p*‐values without consideration of effect sizes or confidence intervals can lead to misinterpretation of the practical significance of findings, multiple testing without appropriate corrections increases the risk of Type I errors (false positives), particularly in studies examining numerous outcomes or subgroups.

#### 15.3.2 Modelling limitations

Inappropriate use of linear models for non‐linear relationships (e.g. dose–response effects) can lead to erroneous conclusions. Failure to account for confounding variables or interactions in statistical models can obscure the true effect of interventions.

#### 15.3.3 Handling of missing data

Inadequate handling of missing data through simplistic approaches like listwise deletion can bias results, especially when the missingness is related to the outcome. The use of more sophisticated methods such as multiple imputation is not instead consistently applied across studies.

#### 15.3.4 Reporting practices

Selective reporting of positive outcomes while neglecting non‐significant or negative results contributes to publication bias. However, insufficient detail about statistical methodologies hinders reproducibility and critical appraisal by other researchers.

In conclusion, the current landscape of obesity research demonstrates a need for methodological rigour and advanced biostatistical approaches. Robust study designs, appropriate sampling strategies, standardized interventions, reliable outcome measures and comprehensive data analysis are paramount for producing valid, reliable and actionable evidence. Addressing these issues will enhance the quality of research and contribute to more effective obesity prevention and treatment strategies.

## AUTHOR CONTRIBUTIONS

FC is project administration and contributed to conceptualization, methodology and wrote the original draft. JPD, JPAI, IJN, GG, LB, LL, SM, GV, THS, MPM, ADC, MK, AG, GB, MFF, IF, NS, GF, PY‐E, JG‐A, EC‐M, VV‐V, J‐MO, DNK, PS, CZ and PP wrote the original draft, Fabrizio Montecucco critically reviewed and edited the manuscript.

## CONFLICT OF INTEREST STATEMENT

Ian J Neeland received consulting fees/speaker fees from Boehringer Ingelheim, Eli Lilly, Novo Nordisk and Bayer. Luca Busetto received payment of honoraria from Lilly, Novo Nordisk, Boehringer Ingheleim, Pfizer and Regeneron as a member of advisory boards, and payment of honoraria for lectures from Rhytms Pharmaceuticals. Luca Liberale is co‐inventor on the international patent WO/2020/226993 filed in April 2020. The patent relates to the use of antibodies which specifically bind IL‐1α to reduce various sequelae of ischaemia–reperfusion injury to the central nervous system. Luca Liberale reports speaker fees from Daiichi‐Sankyo outside the submitted work and has received funding from the Novartis Foundation for Medical‐biological Research (unrelated to this work). Gema Frühbeck received payment of honoraria from Lilly, Novo Nordisk, Regeneron and AstraZeneca as a member of advisory boards, and payment of honoraria for lectures as member of the OPEN Spain Initiative. Verónica Vázquez Velázquez has an unpaid position of Chair of the non‐profit organization Obesidades Mexico, declares honoraria for expert advice on educational projects and campaigns related to obesity from Novo Nordisk and Ely Lilly, honoraria and travel support for lectures, presentations in conferences and educational events from Novo Nordisk, Eli Lilly, Merck and Boehringer Ingelheim, support for attending academic events from Novo Nordisk and World Obesity Federation, support for participation on advisory boards from Novo Nordisk, Eli Lilly and Boehringer Ingelheim, and grants for Obesidades for carrying out an awareness campaign, for a research project and for social activities from Novo Nordisk. Paolo Sbraccia received payment of honoraria from Lilly, Novo Nordisk, Boehringer Ingheleim, Pfizer, Amryt (Chiesi) as a member of advisory boards and payment of honoraria for lectures from Lilly, Novo Nordisk, Amryt (Chiesi). Emma Chávez Manzanera reports honoraria for lectures, presentations and educational events from Novo Nordisk, Merck, Boehringer Ingelheim and Silanes; advisory board honoraria from Abbott, Eli Lilly and Merck. Andreas Geier reports support from the IMI2 LITMUS project; research grants from Novartis, Falk and Intercept; consulting or speakers fees from Abbvie, Advanz, Albireo, Alexion, AstraZeneca, Bayer, BMS, Boehringer, Burgerstein, CSL Behring, Eisai, Falk, Gilead, Heel, Intercept, Ipsen, Merz, MSD, Novartis, NovoNordisk, Orphalan, Pfizer, Roche, Sanofi‐Aventis.; and travel/meeting supports from Intercept, Gilead, Abbvie and Falk. The other authors declared no conflict of interest.

## Data Availability

No new data were generated or analysed in this study. This is a review article based on previously published data and literature.
